# Functional expansion of a TCA cycle operon mRNA by a 3′ end-derived small RNA

**DOI:** 10.1093/nar/gky1243

**Published:** 2018-12-12

**Authors:** Masatoshi Miyakoshi, Gianluca Matera, Kanako Maki, Yasuhiro Sone, Jörg Vogel

**Affiliations:** 1 Department of Infection Biology, Faculty of Medicine, University of Tsukuba, 305-8575 Tsukuba, Japan; 2 Department of Biotechnology, Akita Prefectural University, 010-0195 Akita, Japan; 3 Center for Food Science and Wellness, Gunma University, 371-8510 Maebashi, Japan; 4 RNA Biology Group, Institute for Molecular Infection Biology, University of Würzburg, D-97080 Würzburg, Germany; 5 Helmholtz Institute for RNA-based Infection Research, D-97080 Würzburg, Germany

## Abstract

Global RNA profiling studies in bacteria have predicted the existence of many of small noncoding RNAs (sRNAs) that are processed off mRNA 3′ ends to regulate other mRNAs via the RNA chaperones Hfq and ProQ. Here, we present targets of SdhX (RybD), an Hfq-dependent sRNA that is generated by RNase E mediated 3′ processing of the ∼10 000-nt mRNA of the TCA cycle operon *sdhCDAB-sucABCD* in enteric bacteria. An *in silico* search predicted *ackA* mRNA, which encodes acetate kinase, as a conserved primary target of SdhX. Through base pairing, SdhX represses AckA synthesis during growth of *Salmonella* on acetate. Repression can be achieved by a naturally occurring 38-nucleotide SdhX variant, revealing the shortest functional Hfq-associated sRNA yet. *Salmonella* SdhX also targets the mRNAs of *fumB* (anaerobic fumarase) and *yfbV*, a gene of unknown function adjacent to *ackA*. Instead, through a slightly different seed sequence, SdhX can repress other targets in *Escherichia coli*, namely *katG* (catalase) and *fdoG* (aerobic formate dehydrogenase). This study illustrates how a key operon from central metabolism is functionally connected to other metabolic pathways through a 3′ appended sRNA, and supports the notion that mRNA 3′UTRs are a playground for the evolution of regulatory RNA networks in bacteria.

## INTRODUCTION

The development of the operon concept was a landmark event in the history of molecular biology. Originally proposed as a regulatory mechanism for the *lac* genes in *Escherichia coli* (1), an operon nowadays tends to be more simply defined as a cluster of genes that are transcribed into a single mRNA molecule. This type of gene organization enables organisms to simultaneously turn on or off a set of structural genes of related function, which can be advantageous when the encoded proteins are involved in the same protein complex, function in common pathways, or share substrates and transporters. Operons are the major gene expression units in bacterial genomes. According to global gene expression studies in diverse organisms (2–4) as well as *in silico* prediction platforms (5,6), typically >60% of a bacterium's genes are part of an operon. However, these numbers refer to protein-encoding cistrons only and largely ignore the possibility that the operon mRNA itself, either in its primary form or upon processing, may also have a protein-independent function (7–9).

Recent evidence for abundant noncoding functions of operon mRNAs has come from studies of two major bacterial RNA binding proteins (RBPs), Hfq and ProQ, in *Escherichia coli* and *Salmonella* Typhimurium. Specifically, the *in vivo* target suites of these RBPs were found to contain dozens of abundant small noncoding RNAs (sRNAs) that are processed off the 3′ end of mRNAs (10–16). Almost half of these sRNAs stem from polycistronic transcripts of operons and are likely to extend or complement the physiological function of the operon's proteins by base pairing with other transcripts. For example, SroC sRNA is made from the *gltIJKL* operon mRNA (7) and acts to promote the decay of GcvB sRNA (17). Here, the operon-derived SroC sRNA is functionally related to the operon's proteins: the *gltIJKL* genes encode an amino acid transporter while GcvB is a major post-transcriptional regulator of amino acid-related genes (18). However, whether such functional relatedness is the rule or the exception requires knowledge of the targets of those many other operon-derived sRNAs that are currently of unknown function.

**Figure 1.**
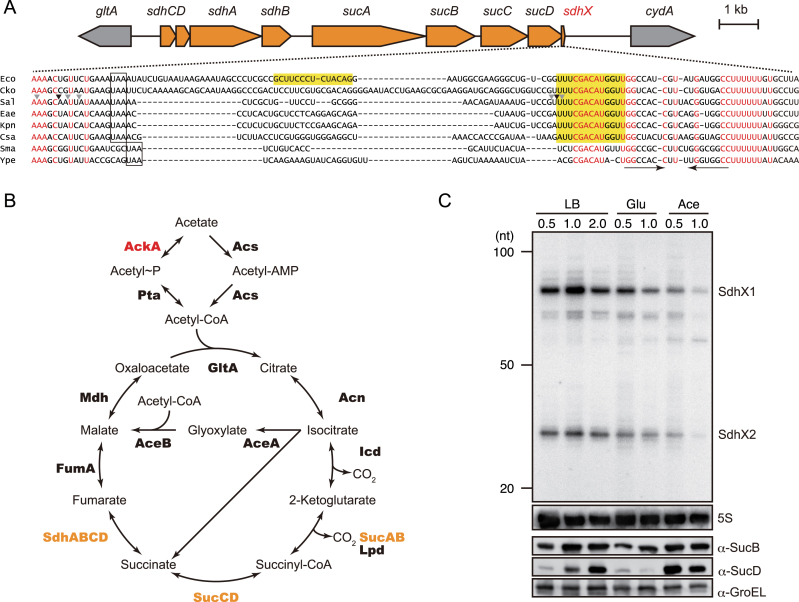
(**A**) Genetic structure of *sdhCDAB-sucABCD* operon and alignment of *sucD* 3′UTRs of selected enterobacterial species. Nucleotide sequences were obtained from the following genomes: Eco, *E. coli* MG1655 (NC_000913); Cko, *Citrobacter koseri* ATCC BAA-895 (NC_009792); Sal, *Salmonella* Typhimurium SL1344 (NC_016810); Eae, *Enterobacter aerogenes* KCTC 2190 (NC_015663); Kpn, *Klebsiella pneumoniae* 342 (NC_011283); Csa, *Cronobacter sakazakii* ATCC BAA-894 (NC_009778); Sma, *Serratia marcescens* FGI94 (NC_020064); Ype, *Yersinia pestis* CO92 (NC_003143). Red letters indicate conserved nucleotides. The stop codons of the *sucD* open reading frame are boxed. The conserved seed region and *E. coli*-specific region complementary to *katG* are highlighted. The Rho-independent terminator is indicated by inverted arrows. RNase E cleavage sites in *Salmonella* identified by (26) are indicated by arrow heads, the major and minor sites of which are in black and grey, respectively. (**B**) Acetate catabolic pathway into TCA cycle. The enzymes encoded on *sdhCDAB-sucABCD* operon are shown in orange. The enzymes regulated by SdhX in *Salmonella* are shown in red. (**C**) Expression profile of TCA cycle proteins and *sucD* mRNA 3′UTR-derived sRNAs. *Salmonella* Typhimurium strain SL1344 was aerobically grown in LB medium or minimal medium supplemented with 0.2% glucose (Glu) or 40 mM acetate (Ace). *Salmonella* cells were collected at the indicated OD_600_ values and whole cell samples and total RNA samples were analyzed on western and northern blots. The size is estimated by DynaMarker RNA Low II ssRNA fragments.

The present work addresses the elusive function of SdhX (previously RybD (15), renamed in agreement with the Gottesman group (19), which is an exceptionally strong candidate of a functional 3′ end-derived sRNA. Originally predicted in a pioneering global screen for Hfq interacting transcripts in *E. coli* (15), SdhX presented as an abundant RNA species from a variable region downstream of the *sdh-suc* genes (Figure 1A). The *sdh-suc* operon encodes three enzyme complexes that catalyse successive reactions in the tricarboxylic acid (TCA) cycle (Figure 1B). It becomes fully expressed during aerobic growth on acetate or fatty acids (20) and displays a complex regulation, involving the activity of different transcription factors, Crp, ArcA/ArcB and RpoS, at the upstream *sdhC* promoter (21–24), internal termination and an additional promoter in the region between *sdhB* and *sucA* (4,25), as well as processing of the ∼10-kb long mRNA by RNase III and RNase E (26–28). Importantly, available data (11,29) suggest that both the expression of SdhX and its strong enrichment with Hfq are conserved in *Salmonella*. However, SdhX’s function and how it might be related to the operon's proteins has remained unknown.

SdhX is of particular interest for additional reasons. Since its shortest form is predicted to be only 38 nucleotides in length (11), a functional sRNA that compact could help to finally obtain a crystal structure of an Hfq–sRNA–mRNA complex (30). Moreover, since the *sdhX* region varies considerably even amongst closely related bacteria (Figure 1A), this sRNA may be an excellent example to understand how an otherwise conserved operon has used a flexible RNA component to adapt its function during microbial evolution. With these considerations in mind, we have predicted and experimentally validated SdhX targets in *Salmonella* and *E. coli*. Besides conserved mRNA targets, we have identified regulation that is specific to one or the other species and arose through either synonymous mutations in the target genes or a mutation in the 3′ UTR of the *sdh-suc* operon. The targets we identify suggest that SdhX connects the *sdh-suc* operon to other central metabolic pathways. Furthermore, our findings support the growing notion that mRNA 3′ UTRs serve as an ‘evolutionary playground’ to generate new regulatory RNAs (31,32).

## MATERIALS AND METHODS

### Bacterial strains and growth conditions

*Salmonella enterica* serovar Typhimurium strain SL1344 (JVS-1574) and *E. coli* strain BW25113 were used as a wild-type strain. The strains used in this study are listed in Supplementary Table S1. Bacterial cells were grown at 37°C with reciprocal shaking at 180 rpm in LB Miller medium (BD Biosciences) or MOPS minimal medium (33), supplemented with 0.2% glucose or 40 mM sodium acetate. Throughout the growth, optical density (OD600) was monitored at every 10 min using OD-MonitorC&T (TAITEC). Where appropriate, media were supplemented with antibiotics at the following concentrations: 100 μg/ml ampicilin (Ap), 50 μg/ml kanamycin (Km) and 20 μg/ml chloramphenicol (Cm).

### Plasmid construction

A complete list of all plasmids and oligonucleotides used in this study can be found in Supplementary Tables S2 and S3. Expression plasmids of SdhX from *Salmonella* and *E. coli*, pLM1 and pLM48, were constructed by cloning the PCR fragment amplified with JVO-7189/JVO-5376 and JVO-7499/JVO-13379, respectively, into pZE12-luc as described previously (34). *Salmonella* SdhX2 expression plasmid pLM30 was constructed by PCR amplification with JVO-12421/JVO-13329 and self-ligation. Translational fusion plasmids based on pXG10-sf and pXG30-sf plasmids were constructed as described previously (34,35). Single-nucleotide mutations were introduced by inverse PCR using overlapping primers followed by DpnI digestion (Supplementary Tables S2 and S3).

### Strain construction

Deletion strains were constructed by the lambda Red system (36). The *sucD* 3′UTRs in between the stop codon and Rho-independent terminator were deleted using pKD4 as a template and primer pairs, JVO-7178/MMO-0182 for *Salmonella* and JVO-13380/MMO-0183 for *E. coli*, respectively. The resulting Km resistant strains were confirmed by PCR and the mutant loci were transduced into appropriate genetic backgrounds by P22 and P1 phages in *Salmonella* and *E. coli*, respectively. To eliminate the resistance genes from the chromosome, strains were transformed with the temperature-sensitive plasmid pCP20 expressing FLP recombinase (36).

Chromosomal single-nucleotide mutants of SdhX were constructed by scar-less mutagenesis through a two-step lambda Red recombination (37). DNA fragments containing a Cm^R^ resistance marker and a I-SceI recognition site were amplified with primer pairs JVO-7178/JVO-12240 using pWRG100 plasmid as a template, and were integrated into the chromosomal *sucD* 3′UTR by lambda Red recombinase expressed from pKD46 (36). The resultant mutants were transformed by pWRG99, and the mutant allele amplified from pLM1 derivatives (Supplementary Table S2) with JVO-7499/JVO-5376 were integrated by the lambda Red recombinase expressed from pWRG99. To eliminate the Cm^R^ I-SceI allele, I-SceI endonuclease was subsequently expressed from the same plasmid and selected the resultant recombinant on LB agar plate supplemented with Ap and 2 μg/ml of anhydrotetracyclin. The successful recombinants were confirmed by Cm sensitivity, PCR, and sequencing.

The 3xFLAG epitope tag at the C-terminus of *ackA* was amplified with primer pairs JVO-13308/JVO-13309 using pSUB11 (38) as a template, and was introduced into the chromosome by the lambda Red system (36). The resulting Km resistant strains were confirmed by PCR and the mutant loci were transduced into appropriate genetic backgrounds by P22 phage.

### GFP fluorescence quantification

Single colonies (triplicates) of *Salmonella* and *E. coli* Δ*sdhX* strains harboring super folder GFP (sfGFP) translational fusions and sRNA expression plasmids were inoculated in 1 ml LB containing Ap and Cm, and were grown overnight at 37°C. 100 μl of the overnight cultures were dispensed in 96-well optical bottom black microtiter plates (Thermo Scientific #165305), and OD600 and fluorescence (excitation at 476 nm and emission at 510 nm, using emission cutoff filter of 495 nm) were measured using SpectraMax M5 (Molecular Devices).

### Western blot analysis

Western blot was performed following a previously published protocol (34). Briefly, bacteria culture was collected by centrifugation for 2 min at 16 100 *g* at 4°C, and the pellet was dissolved in 1× protein loading buffer to a final concentration of 0.01OD/μl. After heating for 5 min at 95°C, 0.002OD of whole-cell samples were separated on 10% TGX gels (Bio-Rad). Proteins were transferred onto a PVDF membrane for 30 min at 10V using a semi-dry blotter in transfer buffer (25 mM Tris, 190 mM glycine, 20% methanol, pH 8.3). Membranes were blocked for 1 h in 1× Block-Pro buffer (Wako Pure Chemical Industries) and rinsed in 1× TBST buffer (20 mM Tris, 150 mM NaCl, 0.1% Tween20, pH 7.6). After blocking, membranes were incubated overnight at 4 °C with monoclonal α-FLAG (Sigma-Aldrich #F1804; 1:5000), polyclonal α-SucB (provided by Kan Tanaka, Tokyo Institute of Technology; 1:10 000), polyclonal α-SucLG1 (Sigma-Aldrich #SAB2700409; 1:1000), polyclonal α-OmpA (1:10 000) or polyclonal α-GroEL (Sigma-Aldrich #G6532; 1:10 000) antibodies diluted in 1xTBST buffer containing 3% BSA or skim milk, and were washed three times for 15 min in 1× TBST buffer. Then membranes were incubated for 1h at RT with secondary α-mouse or α-rabbit HRP-linked antibodies (Cell Signaling Technology #7076 or # 7074; 1:5000) diluted in 1× TBST buffer containing 3% BSA or skim milk, and were washed three times for 15 min in 1× TBST buffer. Chemiluminescent signals were developed using Amersham ECL Prime reagents (GE Healthcare), visualized on LAS4000 or Imager 600 (GE Healthcare) and quantified using Image Quant TL software (GE Healthcare).

### Northern blot analysis

Bacterial culture was mixed with 0.2 (v/v) of stop solution (95% ethanol and 5% phenol) and immediately frozen. Total RNA was isolated using the TRIzol reagent (Invitrogen), treated by TURBO DNase (Invitrogen), and precipitated by cold ethanol. RNA was quantified using NanoDrop One (Invitrogen). 5 μg of total RNA was denatured at 95°C for 5 min in RNA loading buffer (95% v/v formamide, 10 mM EDTA, 0.1% w/v xylene cyanole, 0.1% w/v bromophenol blue) and separated by gel electrophoresis on 8% polyacrylamide/7 M urea gels in 1xTBE buffer for 3h at 250V using Biometra Eco-Maxi system (Analytik-Jena). RNA was transferred from the gel onto Hybond-N+ nylon membrane (GE Healthcare) by electroblotting for 1h at 50V using the same system. The membrane was crosslinked by 120 mJ/cm^2^ UV light. After prehybridization in Rapid-Hyb buffer (Amersham), a [^32^P]-labeled probe was hybridized at 42°C overnight. Membrane was washed in three 15-min steps in 5× SSC/0.1% SDS, 1× SSC/0.1% SDS and 0.5x SSC/0.1% SDS buffers at 42°C. Oligonucleotides MMO-0315 and MMO-0317 for SdhX, JVO-0322 for 5S rRNA and JVO-13619 for tRNA^Pro^ were 5′-end-labeled with [^32^P]-γ-ATP by T4 polynucleotide kinase and purified over G25 columns (GE Healthcare). The MspI-digested pUC19 dsDNA (Fermentus) or DynaMarker RNA Low II ssRNA fragments (BioDynamics Laboratory Inc.) was similarly labeled with [^32^P]-γ-ATP by T4 polynucleotide kinase and was used as a size marker. Signals were visualized on Amersham Typhoon scanner and quantified using Image Quant TL software (both GE Healthcare).

## RESULTS

### Expression characteristics and major forms of SdhX

Signals from the *sdhX* region after the *sucD* stop codon are readily detectable on northern blots under several growth conditions of *Salmonella* (Figure 1C, upper part). The two most abundant SdhX species were designated SdhX1 and SdhX2. The steady-state levels of SdhX were highest in *Salmonella* grown in rich medium to late exponential phase (OD_600_ of 1), and generally much lower in a minimal growth medium containing glucose or acetate as the sole carbon source. Interestingly, SdhX levels were rather lower during growth on acetate than on glucose and their accumulation does not necessarily correlate with the levels of proteins from the same operon, such as SucB or SucD (Figure 1C, lower part), which increases the previously reported complexity of output from the *sdhCDAB-sucABCD* operon (25,27,39).

### Biogenesis of SdhX by RNase E dependent operon mRNA processing

In principle, SdhX could be generated by two different mechanisms: by operon-independent transcription starting within *sucD* and ending at the operon's terminator, analogously to *E. coli* MicL sRNA whose promoter lies within the *cutC* coding region (40); or by co-transcription and mRNA 3′ processing of the *sdh-suc* operon. In favour of the former mechanism was the observed incongruent accumulation of SucD and SdhX (Figure 1C). However, since the 5′ positions of SdhX1 or SdhX2 are preceded by neither a known transcription start site (4,29,41) nor a conserved promoter-like element (Figure 1A), biogenesis through mRNA processing seemed more likely. In addition, our previously published global predictions of putative cleavage sites of the major endonuclease RNase E in the *Salmonella* transcriptome (26) suggested processing upstream of the *sucD* stop codon and in the 3′UTR that matched the 5′ ends of SdhX1 (87 nt) and SdhX2 (38 nt), respectively (Figure 1A). The 5′ status and sizes of these two SdhX species are fully supported by other diverse RNA-seq data sets we have obtained for *Salmonella* Typhimurium strain SL1344 (12,26,42,43).

**Figure 2.**
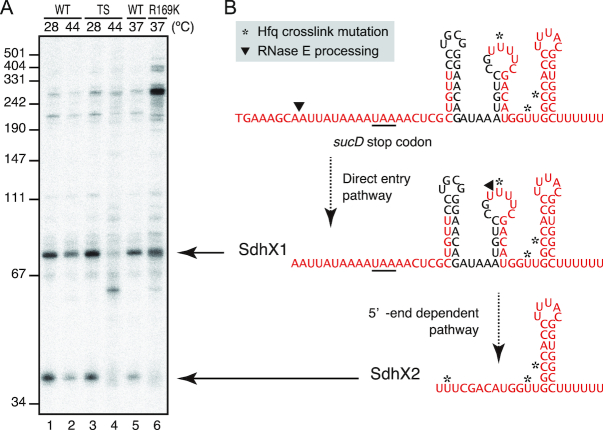
SdhX is processed by RNase E. (**A**) *Salmonella rne*+ (WT: lanes 1–2) and *rne3071* (TS: lanes 3–4) strains were grown to OD_600_ of 0.5 at 28°C, split into two flasks, and further incubated at either 28°C (lanes 1, 3) or 44°C (lanes 2, 4) for 30 min. *Salmonella rne*+ (WT: lane 5) and *rneR169K* (R169K: lane 6) strains were grown to OD_600_ of 0.5 at 37°C. The size is estimated by pUC19 MspI dsDNA fragments. (**B**) Predicted secondary structures of SdhX1 and SdhX2. Hfq-bound regions identified by CLIP-seq analysis (12) are indicated by red letters, and the mutations induced by crosslinking to Hfq are highlighted in yellow. See also Supplementary Figure S1.

To corroborate the hypothesis that SdhX biogenesis occurs by RNase E-mediated operon mRNA processing, we used *Salmonella* strain *rne3071* which expresses a temperature-sensitive RNase E protein (44,45). Comparing to the *Salmonella rne*^+^ control strain, the *rne3071* mutant at non-permissive temperature (44°C) exhibited a clear reduction of both SdhX species (Figure 2A, lanes 1–4). This indicates that mRNA processing by RNase E is essential to make SdhX.

To better understand the order of processing events, we used a *Salmonella rneR169K* strain (17) with a 5′ sensor-deficient RNase E which stops the enzyme preferring processed 5′-monophosphate over primary 5′-triphosphate transcripts (46). For example, this strain is deficient in 5′ end-dependent processing of 5S rRNA but remains proficient in direct entry-mediated processing of tRNA^Pro^ (Supplementary Figure S1). As shown in Figure 2A, lanes 5–6, the 5′ sensor mutation hardly affected the levels of SdhX1 while it did diminish the SdhX2 signal. In addition, a ∼300-nt long mRNA processing fragment which likely contains a good part of the *sucD* coding region accumulated. We interpret these observations to mean that SdhX2 is processed from SdhX1 by RNase E via the 5′ end dependent pathway, whereas SdhX1 can be generated by direct entry of RNase E or other ribonucleases (Figure 2B).

### Predicted mRNA targets of SdhX in *Salmonella*

Hfq-dependent sRNAs repress or activate mRNA targets by a variety of mechanisms (47), with a major mechanism being that they base-pair near the ribosome binding site (RBS) of mRNAs to compete with 30S ribosome binding and therefore translation initiation (48,49). Most commonly, this involves the structurally accessible, conserved seed region of an sRNA, a candidate of which in the present case of SdhX is the UUCGACAUGGU stretch that precedes the terminator stem–loop of this sRNA (compare Figures 1A and 2B).

To predict conserved mRNA targets, we ran *in silico* searches with the comparative CopraRNA algorithm (50) in six enterobacterial genomes, using the SdhX2 sequence as query (Supplementary Figure S2A). Interestingly, of several conserved mRNA targets predicted in this search, almost all would be recognized through the assumed seed sequence of SdhX (Supplementary Figure S2B and C). Most of them would involve complementarity of the SdhX seed with the AUG start codon and a UCG codon (serine) at the second mRNA position.

**Figure 3.**
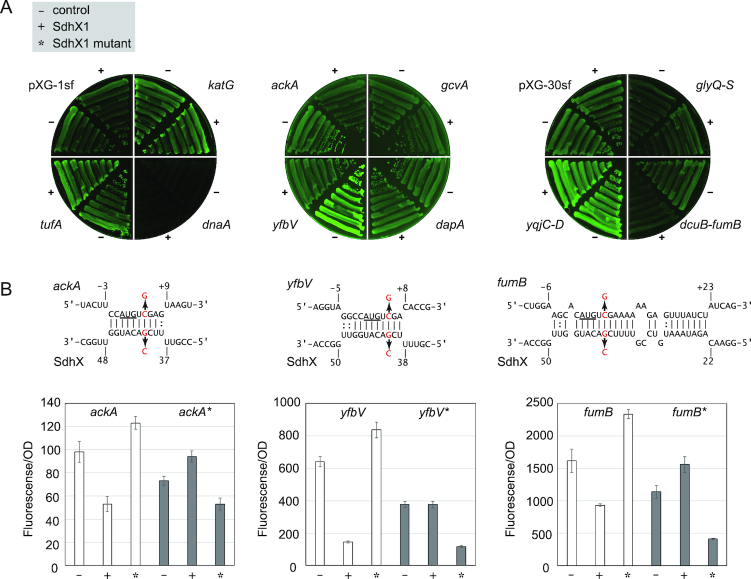
The *ackA, fumB* and *katG* mRNAs are regulated by *Salmonella* SdhX. (**A**) The 5′UTRs or intergenic regions of candidate target mRNAs were cloned into pXG-10sf and pXG-30sf vectors, respectively. *Salmonella* Δ*sdhX* strain was transformed by pXG derivative plasmids along with pJV300 control vector (-) or SdhX1 expression plasmid (+). GFP expression on the plate was visualized by LAS4000 imager. (**B**) Predicted interactions of *Salmonella* SdhX1 with target mRNAs. Mutated nucleotides (G41C in *sdhX* and C5G in target mRNAs) were indicated by red letters. The start codon of target mRNAs is underlined. (B) SdhX regulates the target mRNAs by base-pairing mechanism. *Salmonella* Δ*sdhX* strain was transformed by combinations of pXG plasmids along with pJV300 control vector (–), SdhX1 expression plasmid (+) or SdhX1 mutant expression plasmid (*) as indicated. GFP expression in the LB liquid medium was quantified by a plate reader and normalized by OD_600_. Since each translational fusion exhibited various GFP intensities, the graphs are shown at different scales. Error bars indicate standard deviations (*n* = 3).

For preliminary evaluation of these predictions, we cloned the top 10 targets as gene fusions to sfGFP in reporter plasmids pXG-10sf or pXG-30sf (35). These fusions were introduced in *Salmonella* together with a ColE1-derivative plasmid that constitutively expressed SdhX1. Of the 10 genes tested, we observed that *ackA, yfbV* and *fumB* were significantly repressed by SdhX1 (Figure 3A). Of note, *ackA* is the first gene of the *ackA*-*pta* operon that encodes the metabolic pathway converting acetate and acetyl-CoA via a high-energy intermediate compound, acetyl-phosphate (acetyl-P) (51). Interestingly, the *ackA-pta* operon is located adjacent to the *yfbV* gene, which encodes a cytoplasmic protein predicted to be involved in chromosome segregation in *E. coli* (52). The third validated target, *fumB* encodes one of the three fumarases that is induced under anaerobic condition. Here, SdhX is predicted to base-pair with the intergenic spacer within the bicistronic *dcuB-fumB* operon.

To confirm that SdhX1 regulates these mRNAs by direct base pairing, complementary mutations were introduced into the two expression plasmids. A G_41_→C mutation in SdhX1 (mutation at the 41st nucleotide after the stop codon of *sucD*) invariably reduced the repression of *ackA, yfbV* and *fumB* (Figure 3B). Importantly, the mutation did not affect the expression levels of SdhX1 and SdhX2 (data not shown). However, a compensatory mutation (C5G; non-synonymous mutation changing the second codon from serine to tryptophan), although slightly decreasing the expression of each of the three GFP translational fusions (Figure 3B), rendered all of them susceptible to regulation by the mutant sRNA. These successful compensatory base pair changes strongly support our prediction that SdhX recognizes the RBS of these mRNAs to cause translational repression.

### SdhX represses *ackA* mRNA during growth on acetate

**Figure 4.**
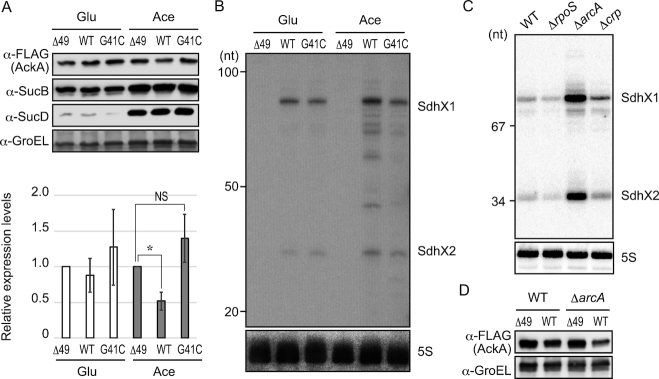
Physiological levels of SdhX repress AckA synthesis during aerobic growth on acetate. (**A**) *Salmonella* chromosomal *sdhX* mutants with C-terminal *ackA*::FLAG fusions (*sdhX* WT, JVS-11249; *sdhX*G41C, JVS-11250; *sdhX*Δ49, MMS-0005) were grown to exponential phase in MOPS minimal medium supplemented with 0.2% glucose (Glu) or 40 mM sodium acetate (Ace). The whole cell samples were analysed by western blot. Expression level of AckA was normalized by that of GroEL, and relative expression levels in *sdhX* WT and G41C strains to that in Δ49 mutant were plotted. Error bars indicate standard deviations (*n* = 5). Asterisk indicates *P* < 0.01. NS indicates not significant (*P* > 0.05). (**B**) RNA samples extracted from the cells grown as in (A) were analysed by northern blot using ^32^P-labelled oligonucleotides for SdhX2 (MMO-0315) and 5S rRNA (JVO-0322). The G41C mutant exhibited weaker signals when MMO-0317 containing the single mismatch was used (data not shown). The size is estimated by DynaMarker RNA Low II ssRNA fragments. (**C**) *Salmonella* strains deleted for transcriptional factors (lane 1, JVS-1574; lane 2, JVS-0673; lane 3, JVS-1227; lane 4, JVS-1626) were grown to exponential phase in LB medium, and analysed by northern blot using MMO-0317 and JVO-0322. A pUC19 MspI dsDNA ladder was used as size marker. (**D**) *Salmonella ackA*::FLAG strains (*sdhX* WT; JVS-11249 and *sdhX*Δ49; MMS-0005) and those in Δ*arcA* genetic background carrying either an intact *sdhX* region (*sdhX* WT; MMS-0015) or a mutant thereof (*sdhX*Δ49; MMS-0014) were grown to exponential phase in LB medium, and analysed by western blot with anti-FLAG and anti-GroEL antibodies.

The reporter assays above confirmed that several of the predicted targets can be regulated by SdhX but did not prove that regulation occurred with physiological concentrations of the sRNA. To establish proof-of-principle for endogenous regulation, we selected *ackA* for its SdhX site is conserved amongst the Enterobacteriaceae. In the absence of a specific antibody for western blot analysis, we rendered the AckA protein detectable through adding a C-terminal FLAG tag in the *Salmonella* chromosome. Likewise, we generated two chromosomal mutations in the *sucD* 3′UTR, either replacing the whole 3′UTR but the Rho-independent terminator with an unrelated FRT sequence (Δ49), or introducing the afore described G_41_→C point mutation into the SdhX seed region. Importantly, these modifications of the 3′UTR did not influence the translation of SucD (Figure 4A), while deletion of the whole 3′UTR significantly reduced SucD protein level and impaired growth on acetate (data not shown).

*Salmonella ackA*::FLAG bacteria with wild-type or the mutated *sdhX* regions were grown on two different primary carbon sources, glucose or acetate. While AckA levels showed no SdhX-specific variation under growth in glucose-containing media, growth on acetate resulted in a 2-fold decrease in AckA level in wild-type *Salmonella* as compared to the Δ49 strain (*P* < 0.01) (Figure 4A). The AckA level exhibited no statistically significant difference between the Δ49 and G_41_→C strains by *t*-test (*P* > 0.05), suggesting that SdhX regulated *ackA* mRNA through the base-pairing mechanism. Interestingly, both the levels of SdhX sRNA and the AckA protein were comparable on both carbon sources (Figure 4B), although in contrast to Figure 1C we observed a slight increase of SdhX during growth on acetate that we attribute to the *ackA*::FLAG genetic background. This indicates that there might be another factor that aids SdhX-mediated regulation in the acetate condition (or prevents it when *Salmonella* is grown on glucose).

Seeking conditions to further increase SdhX levels from the chromosome to test an expected higher repression of AckA, we genetically inactivated three predicted transcriptional regulators of the *sdh-suc* operon, based on knowledge from *E. coli* (22–24). Overexpression of SdhX was indeed observed in a *Salmonella* strain inactivated for the aerobic/anaerobic response regulator protein ArcA during exponential growth in LB medium (Figure 4C). In this background, AckA levels showed the expected further reduction (by 3-fold; *P* < 0.05) but only if s*dhX* was intact, i.e., ArcA-dependent regulation was not seen in the Δ49 mutant lacking functional SdhX (Figure 4D). Altogether, these condition-specific regulations argue that the *ackA* mRNA is a physiologically relevant target of SdhX.

### Target divergence between *Salmonella* and *E. coli*

**Figure 5.**
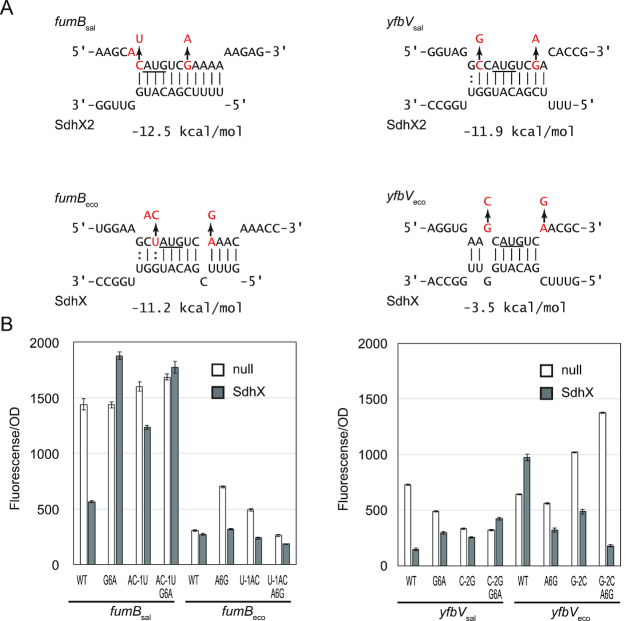
*E. coli fumB* and *yfbV* are not regulated by SdhX due to mutations around their start codons. (**A**) Difference in *fumB*-SdhX and *yfbV*-SdhX interactions between *Salmonella* and *E. coli*. The start codon is underlined. Exchanged nucleotides were indicated by red letters. Changes in free energy (Δ*G*°) upon basepairing are indicated below the interactions. (**B**) *Salmonella* Δ*sdhX* strain was transformed by combinations of mutant pXG plasmids along with pJV300 control vector (null) or SdhX expression plasmid (SdhX). GFP expression was quantified and normalized by OD_600_. Error bars indicate standard deviations (*n* = 3).

As powerful experimental alternatives to *in silico* target finders, recently developed *in vivo* methods have predicted sRNA targets globally in *E. coli* through sequencing RNA-RNA hybrids that copurify with Hfq (13) or RNase E (53). Interrogating the available *E. coli* RIL-seq data (13), we observed on the one hand that the *sucD* 3′ region indeed formed hybrids with many other transcripts including the *ackA* mRNA; on the other hand, hybrids with *fumB* and *yfbV* as observed in *Salmonella* were missing (Supplementary Table S4). In the latter two cases, synonymous nucleotide changes (UCG in *Salmonella*, UCA in *E. coli*) in the serine codon that follows the start codon would lead to slightly altered SdhX-target hybrids in the two organisms (Figure 5A). Interestingly, in the case of *fumB* the difference in predicted duplex strength (change in free energy, Figure 5A) was seemingly negligible, but translational fusions of the *E. coli fumB* and *yfbV* mRNAs were refractory to either *Salmonella* SdhX (Figure 5B) or *E. coli* SdhX (data not shown), contrasting with the 2–4 fold repression of the *Salmonella* fusions.

To test whether these subtle mutations could explain the observed resistance to regulation by SdhX, we changed the corresponding nucleotides of *Salmonella* target mRNAs to those of *E. coli* and *vice versa*. The repression of *fumB*_sal_ and *yfbV*_sal_ by SdhX was strongly abrogated by a synonymous G_6_→A mutation, whereas an inverse A_6_→G mutation rendered *fumB*_eco_ and *yfbV*_eco_ susceptible to SdhX (Figure 5B). In the case of *yfbV*_sal_, however, the G_6_→A change in the second codon did not fully abolish the repression by SdhX. Therefore, the nucleotides upstream of *fumB* and *yfbV* start codon were also mutated (Figure 5A). Both a AC_-1_→U mutation of *fumB*_sal_ and a C_-2_→G mutation of *yfbV*_sal_ substantially reduced the repression level, and fully abrogated it when combined with the G_6_→A mutation. In contrast, the G_-2_→C mutation in *yfbV*_eco_ conferred repression by SdhX and this was further strengthened by the C_-2_→G/G_6_→A double mutation (Figure 5B). These results suggest that although the seed sequence of SdhX is conserved between *E. coli* and *Salmonella* (Figure 1A), in *E. coli* the regulation of the *fumB* and *yfbV* mRNAs was lost due to synonymous mutations around their start codons.

**Figure 6.**
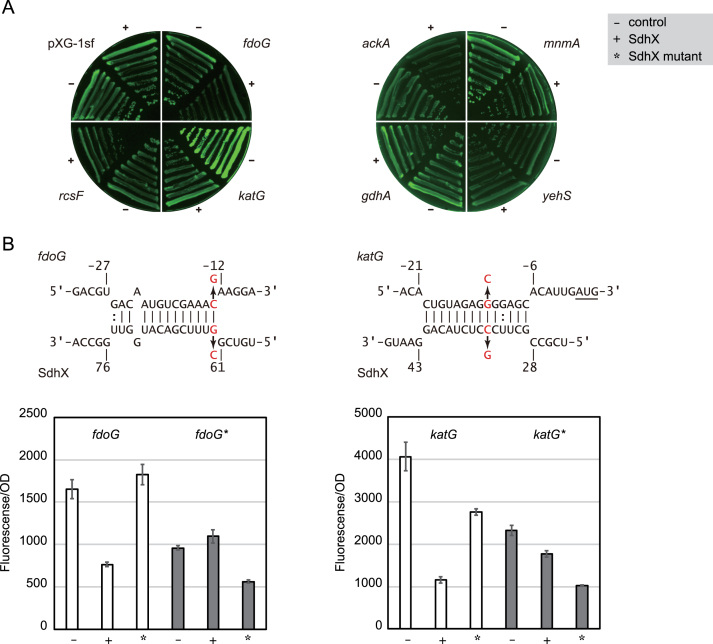
The *fdoG* and *katG* mRNAs are regulated by *E. coli* SdhX. (**A**) The 5′UTRs were cloned into pXG-10sf (35). *E. coli* Δ*sdhX* strain was transformed by pXG derivative plasmids along with pJV300 control vector (–) or SdhX expression plasmid (+). GFP expression (left) and colony densities (right) were visualized by LAS4000 imager. (**B**) Predicted interactions of *E. coli* SdhX with *fdoG* and *katG* mRNAs. Mutated nucleotides were indicated by red letters. The start codon of *katG* is underlined. *E. coli* Δ*sdhX* strain was transformed by combinations of pXG plasmids along with pJV300 control vector (–), SdhX (+) or SdhX G62C or C34G mutant expression plasmid (*) as indicated. Since each translational fusion exhibited various GFP intensities, the graphs are shown at different scales. Error bars indicate standard deviations (*n* = 3).

Conversely to pointing to loss of regulation, the RIL-seq data also listed putative *E. coli* mRNA targets not predicted by the conservation-based bioinformatics approach (Supplementary Table S4), several of which were predicted by the IntaRNA program (54) to interact with SdhX (Supplementary Figure S3). Constructing fusions for seven of these mRNAs, we were able to demonstrate regulation by the *E. coli* SdhX sRNA in *E. coli* itself for three of these putative targets, namely *ackA, fdoG* and *katG* (Figure 6A).

*fdoG* is the first gene of *fdoGHI* operon encoding aerobic formate dehydrogenase (55) and is the most highly enriched RNA fragment with SdhX *in vivo* (13). *fdoG* regulation was abolished by SdhX G_62_→C mutation (at the 62nd nucleotide from the end of *sucD* CDS) (Figure 6B, left). In line with this result, *Salmonella* SdhX (in which G_62_ of *E. coli* SdhX is a U; Figure 1A) failed to repress *fdoG* (data not shown) despite a strong conservation of the *fdoG* translation start region. However, a complementary mutation of *fdoG* C_-13_→G restored the regulation by SdhX G_62_→C (Figure 6B, left), substantiating the prediction that SdhX represses *fdoG* through base pairing. The other new target, *katG*, encodes a bifunctional catalase-peroxidase (56). Interestingly, its Shine-Dalgarno sequence was predicted to base-pair with the variable region between *sucD* CDS and the conserved seed region of SdhX; in other words, upstream of the conserved SdhX2 sequence (Figure 6B, right). Compensatory point mutations demonstrated that SdhX represses *katG* translation using this non-conserved region (Figure 6B, right). This *katG*-targeting region of SdhX is absent in *Salmonella* (Figure 1A), and *katG* was not repressed by SdhX in *Salmonella* (Figure 3A).

### The minimal 38-nucleotide SdhX2 sRNA is a functional regulator

**Figure 7.**
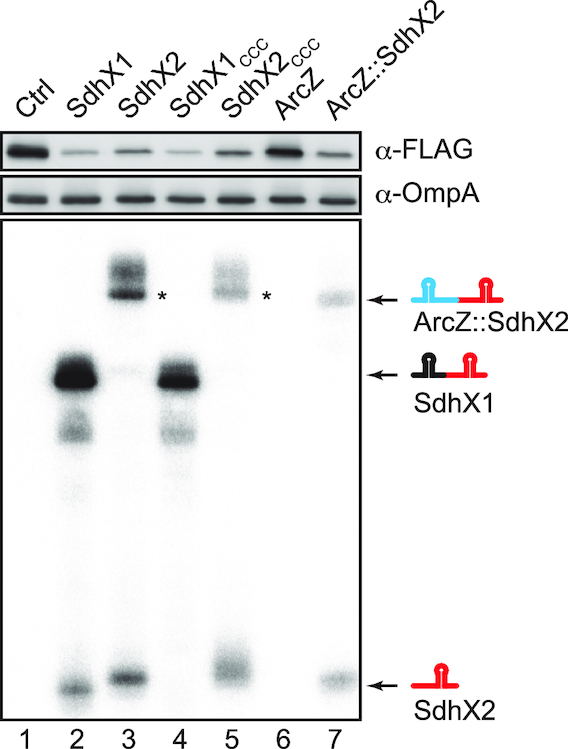
Processing of SdhX2 is not necessary for target regulation. *Salmonella* Δ*sdhX* mutant with C-terminal *ackA*::FLAG fusion was transformed by plasmids expressing respective sRNAs (lane 1: pJV300, lane 2: pLM1, lane 3: pLM30, lane 4: pLM34, lane 5: pLM35, lane 6: pJU-19, lane 7: pLM32). The slightly larger size of SdhX2 when expressed as a primary transcript (lane 3) may result from transcription starting at the –1 instead of the +1 position of the constitutive promoter used here. That is, SdhX2 starts with four uridines which are far from ideal for transcription initiation. Indeed, primer extension analysis revealed an extra nucleotide at the 5′ end of SdhX2 expressed from this plasmid (data not shown). The asterisk indicates a putative read-through product, whose transcription terminates at downstream *rrn*T1 in the vector (34).

The strong variation amongst *sdhX* sequences makes it difficult to settle on the actual regulator. Our results so far had shown that the conserved SdhX2 region was required for the regulation of most targets, but was it also sufficient? To address this, we compared *ackA* mRNA repression upon overexpression of one or the other SdhX species and found almost equal repression by SdhX1 and SdhX2, despite the fact that SdhX2 seemed to be less abundant (Figure 7, lanes 1–3). This suggested that the terminal 38-nt SdhX2 sRNA carries all the information to repress at least this conserved target. Nonetheless, when we interfered with RNase E processing of SdhX1 into SdhX2 by introducing a UUU→CCC mutation at the processing site (Figure 1A), target regulation was still observed (Figure 7, lanes 4 and 5). This result suggests that RNA processing which leaves a 5′ monophosphorylated SdhX2—a 5′ end status that would potentially favour RNase E-mediated target mRNA degradation (57)—is dispensable for activity, at least under a condition of overexpression.

Finally, to be able to assess the potency of SdhX2 in its natural form, i.e. its 5′ monophosphorylated form (as compared to the 5′ triphosphorylated form as the product of transcription; lane 3, Figure 7), we fused its sequence to the upstream part of ArcZ, an sRNA that is similarly matured by RNase E (26,58). This chimeric ArcZ::SdhX2 sRNA construct in which the SdhX2 region follows at the ArcZ processing site effectively expressed a 5′ monophosphorylated SdhX2 (Supplementary Figure S4). And while the wild-type ArcZ had no effect on AckA, this chimeric sRNA repressed AckA synthesis to the same degree as if it was released from SdhX1 (Figure 7, lanes 6 and 7). This result supports a view that the conserved 38-nt SdhX2 part is a fully functional Hfq-dependent sRNA, with the rest of the 3′UTR of *sucD* being variable extensions. Importantly, previous CLIP-seq analysis (12) inferred at least three Hfq contacts in SdhX from UV crosslinking-induced mutations (Figure 2B), suggesting contacts with both internal and 3′-terminal uridines. We therefore expect an Hfq-SdhX2 complex to be very compact and rigid, which could help to overcome the difficulty in crystallization-based analysis of Hfq-sRNA complexes in the past (30).

## DISCUSSION

Hfq and its associated sRNAs have increasingly been found to play important roles in modulating nutrient uptake as well as primary and secondary metabolic pathways in enteric bacteria (59,60), and as part of this, the *sdhCDAB-sucABCD* operon mRNA has emerged as a prominent target of post-transcriptional control (Supplementary Figure S5A). No fewer than three sRNAs, i.e. RyhB, Spot42 and RybB, have been shown to target *sdhC* in *E. coli* (61,62). In addition, the CopraRNA algorithm predicts conserved interactions of the CyaR, FnrS and RyhB sRNAs with the third cistron, *sdhA* (50). The same study experimentally validated a predicted base pairing of the Spot42 sRNA with the 5′ end of *sucC* (50). Notably, Spot42 is induced by glucose (63,64), which offers an explanation for the strong repression of SucD relative to SucB during growth on glucose observed here (Figure 1C). By contrast, the present work in *Salmonella* shows that this long operon mRNA with a central function in primary metabolism is not only a target but also an active regulator in the Hfq network of enteric bacteria. Complementary studies of SdhX in *E. coli* by others (19) further highlight some of the similarities and differences in SdhX regulation in these closely related organisms.

### *trans* regulation via the 3′ ends of metabolic mRNAs

Intrinsic transcription terminators found at the 3′ end of many operon mRNAs are composed of a stem-loop structure followed by a uridine-rich stretch, and are preferred targets of Hfq (65,66). Add an endonucleolytic 3′ cleavage event, which is common in both monocistronic and polycistronic mRNAs (26), and a potential seed sequences, a new Hfq-dependent sRNA may be born (31,32).

Regulatory base pairing functions have already been demonstrated for several mRNA 3′end-derived sRNAs (11,13,17,40,67–72). Most of these sRNAs represent highly conserved 3′UTRs, with the extreme being the CpxQ sRNA whose sequence is far more conserved than the coding region of the parental *cpxP* mRNA (68). By contrast, SdhX exhibits a remarkable lack of conserved nucleotides with the exception of its core seed region (CGACAU) that is complementary to the first two codons of *ackA* and its terminal uridine stretch (Figure 1A). We argue that this type of sRNA is particularly informative as to how regulators continue to evolve in the Hfq network after manifestation of a founding target, here most likely *ackA*.

Assuming *ackA* to be the founding target of SdhX, it is remarkable that most of the additional targets confirmed are similarly involved in carbon flow and respiration. This poses the question whether other operon mRNAs for metabolism have had their functions expanded through 3′ end-derived sRNAs. Indeed, an analysis of 127 regions in mRNA 3′UTRs that are bound by Hfq in *Salmonella* (12) readily predicts several additional strong candidates for cross-connection of metabolic pathways. For example, the *glnA* mRNA encoding glutamine synthetase processes STnc800 from its 3′UTR (73) (Supplementary Figure S5B). *In silico* analysis identified potential base pairing between STnc800 and the intergenic region between *sdhB* and *sucA*. A repression of *sucA* by STnc800 may serve to maintain C/N balance under nitrogen limitation which is when the *glnA* mRNA is most highly expressed. This prediction supports the idea that metabolic mRNAs extend their functions through riboregulators released from their 3′ ends.

### Physiological meaning of regulation by SdhX

We have shown that SdhX regulates the acetate kinase gene *ackA*, for which post-transcriptional regulation is reported for the first time in *Salmonella* (this study) and *in E. coli* by others (19). AckA activates acetate into a high-energy intermediate acetyl-P using ATP (Figure 1B). Acetyl-P is converted into acetyl-CoA by phosphotransacetylase (Pta), an enzyme encoded by the same *ackA-pta* bicistronic mRNA. To utilize acetate as the sole source of carbon, the AckA–Pta pathway is the predominant route when the concentration of acetate is high (>25 mM), while acetyl-CoA synthetase (Acs) is required for growth when acetate is low (<10 mM) (74). As AckA synthesis is repressed ∼2-fold by SdhX during growth on 40 mM acetate (Figure 4A), we expected that an inactivation of *sdhX* would alter bacterial growth. While we have not observed a growth difference with *Salmonella* even when the *sdhX* and *acs* loci inactivated together (Supplementary Figure S6), mutations in *sdhX* that abolish regulation of *ackA* strongly alter the growth behaviour of *E. coli* when acetate is the sole available carbon source (19).

The lack of a robust phenotype as opposed to a clear conservation of the SdhX-*ackA* base pairing interaction may be due to the previously noted robustness of metabolism in this organism (75). Yet, it also echos previous observations with 3′ end-derived sRNAs where strong conservation of the regulatory interaction contrasted with the absence of strong physiological consequences after mutating this region (17,40,68–69). This demands for both, more sensitive detection methods and more complex growth conditions, in order to understand the immediate consequence of SdhX activity. For example, PinT is a conserved sRNA of *Salmonella* that is highly induced when these bacteria replicate inside eukaryotic cells. While standard virulence assays failed to detect a measurable phenotype, profiling the transcriptomes of both *Salmonella* and infected host cells in tandem showed a pervasive function of the PinT sRNAs as a post-transcriptional timer of virulence gene expression (43). Similar approaches applied to transition phases of specific carbon source availability, as pioneered in functional analysis of the *E. coli* Spot42 sRNA (64) may be necessary to fully understand the physiological contribution of SdhX. Potential leads also include the facts that the reversible AckA–Pta pathway mediates excretion of acetate and generates ATP in the overflow metabolism or during anaerobic growth (51), and that *ackA-pta* operon impacts *Salmonella* virulence through SPI1 expression by modulating excreted short-chain fatty acids (SCFAs) (76,77). Since expression of Pta was not significantly altered even when SdhX was overexpressed (data not shown), repression of the first gene in the operon results in discoordination of the metabolic pathway, which may result in accumulation of acetyl-P and non-enzymatic acetylation of many proteins (78–80).

One of the additional target in *Salmonella*, the *yfbV* gene is located adjacent to the *ackA-pta* operon, but its role in the central metabolism is unknown. The *yfbV* transcript is overlapped with the divergent operon mRNA since one of its transcriptional start sites is located in the *ackA* CDS (29), implying that *yfbV* mRNA is involved in the regulation of *ackA-pta* operon in *cis*. Another verified target in *Salmonella* is the *fumB* gene encoding one of the three fumarases that is induced in anaerobic conditions under the control of ArcA and Fnr (81). On the contrary, since the *sdhC* promoter is repressed by ArcA and Fnr (24), we could not detect SdhX sRNAs when *Salmonella* was anaerobically grown in LB medium. Physiological regulation of *fumB* by SdhX might occur only during a shift from aerobiosis to anaerobiosis. *In E. coli*, both the additional targets *fdoG* and *katG* are involved in tolerance to oxidative stress (56,82), in line with an increased sensitivity to hydrogen peroxide by SdhX overexpression in *E. coli* (19). The environment of SdhX at work has apparently been altered between the two enterobacteria depending on oxygen availability.

### Implications for target predictions

The SdhX targets identified here highlight both, the strengths and weaknesses of *in silico* predictions. Upon querying the highly conserved 38-nucleotide SdhX2 sequence, we were able to experimentally confirm 3 of the top 10 predicted targets in *Salmonella*, among which *fumB* and *yfbV* had not been predicted by the *E. coli* RIL-seq data (13). When CopraRNA was run on SdhX from *E. coli* strains only, *katG* was the top candidate, likely because its hybrid with SdhX is more stable than the SdhX-*ackA* interaction (Supplementary Figure S3). However, it is puzzling that most of the SdhX partners in the RIL-seq data (Supplementary Table S4) would not be predicted by CopraRNA or their expression levels were unaffected when tested with reporter fusions (Figure 6A). It remains an intriguing possibility that some of these potential interactors function as RNA sponges, titrating SdhX (83).

Mutational analysis demonstrated that the synonymous mutation in the second codon of *fumB* and *yfbV* was critical for regulation by SdhX where a single nucleotide mutation G_6_→A abrogated the base-pairing with the seed region of SdhX2 located at its 5′ end (Figure 5). Moreover, a wobble base-pairing with U_-1_ of *fumB*_eco_ was one of the reasons why it was not regulated by SdhX in *E. coli* (*fumB*_eco_U-1AC; Figure 5B). In contrast, *E. coli* SdhX acquired regulation of *fdoG* by U→G mutation at the SdhX2 processing site (Figures 1A and 6B). These results are consistent with the case of regulation of *Salmonella* effector proteins by SgrS (84) and suggest that mismatches and G:U pairs are critical for differentiation of post-transcriptional regulation among bacterial species.

The 14-base pair nearly perfect SdhX-*katG* duplex is found only in the *Escherichia/Shigella* clade, but those regions outside the SdhX seed is not predicted to base pair with *katG* in the other enterobacterial species (Figure 1A). Interestingly, Ribo-seq analysis identified *fumB, fdoG* and *katG* as RyhB targets in *E. coli* (85), so these mRNAs may be repeatedly sampled as targets as the Hfq regulatory network evolves. There is a growing tool box for target predictions, running the gamut from *in silico* algorithms (50,86) to experimental searches for either individual sRNAs or in global interactomes (13,87–90), complemented by growing information about where Hfq and RNase E bind in bacterial transcriptomes (12,14,26,53). Running these targets predictions in a comparative manner for representative enteric bacteria seems promising to achieve an understanding of how 3′ UTRs evolve into regulatory RNA molecules.

## Supplementary Material

Supplementary Data
