# Supplementary material for: Functional expansion of a TCA cycle operon mRNA by a 3′ end-derived small RNA
Source: Nucleic Acids Res. 2018 Dec 12;47(4):2075–88. doi: 10.1093/nar/gky1243 (PMC6393394; doi:10.1093/nar/gky1243)
Supplement: Supplementary Data [file gky1243_supplemental_files.zip › Supplementary_revised2.pdf]

Figure S1
Miyakoshi et al., 2018

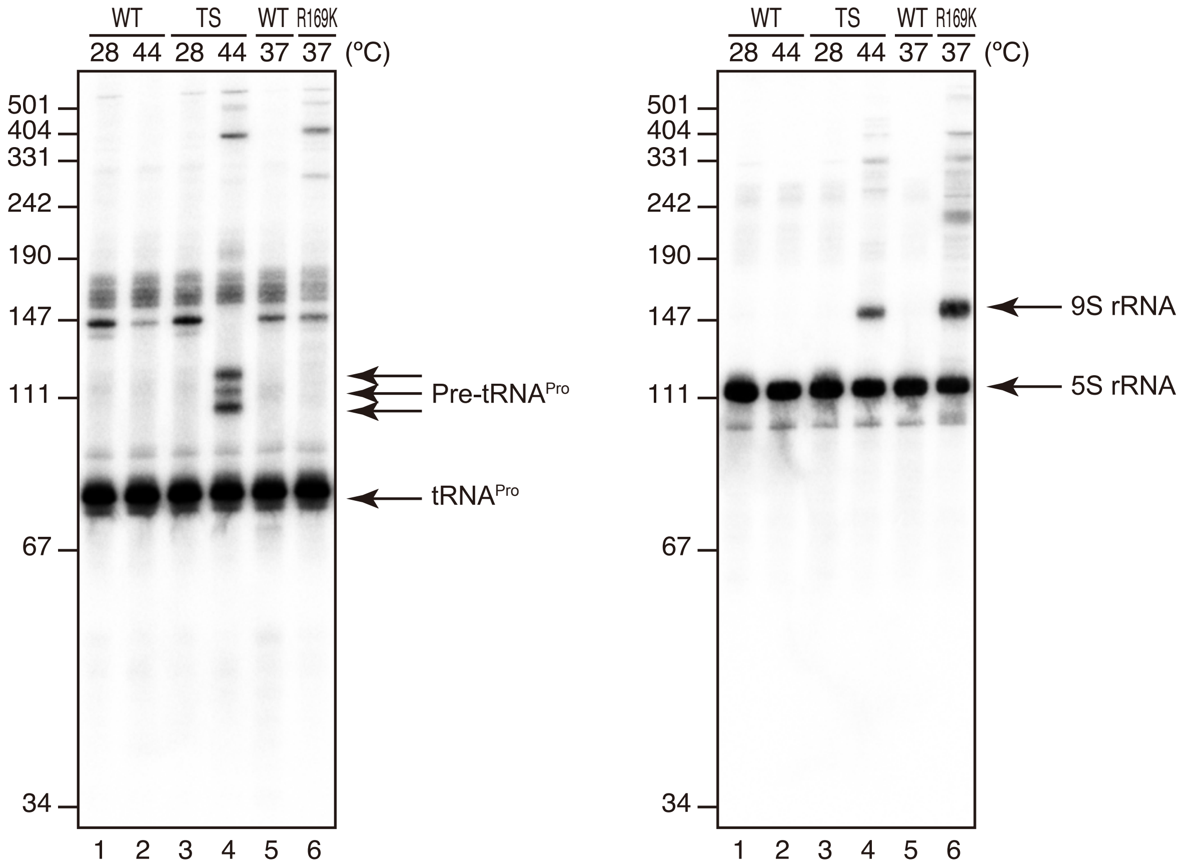

Fig. S1. Processing of 5S rRNA and tRNA-Pro in RNase E mutants.

The same blot as Fig. 3 was re-hybridized with JVO-13619 for tRNA-Pro (left) and JVO-0322 for 5S rRNA (right). *Salmonella rne*$^{+}$ (WT: lanes 1-2) and *rne3071* (TS: lanes 3-4) strains were grown to OD600 of 0.5 at 28ºC, split into two flasks, and further incubated at either 28ºC (lanes 1, 3) or 44ºC (lanes 2, 4) for 30 min. *Salmonella rne*$^{+}$ (WT: lane 5) and *rneR169K* (R169K: lane 6) strains were grown to OD600 of 0.5 at 37ºC. The size is estimated by pUC19 MspI dsDNA fragments.

Figure S2
Miyakoshi et al., 2018

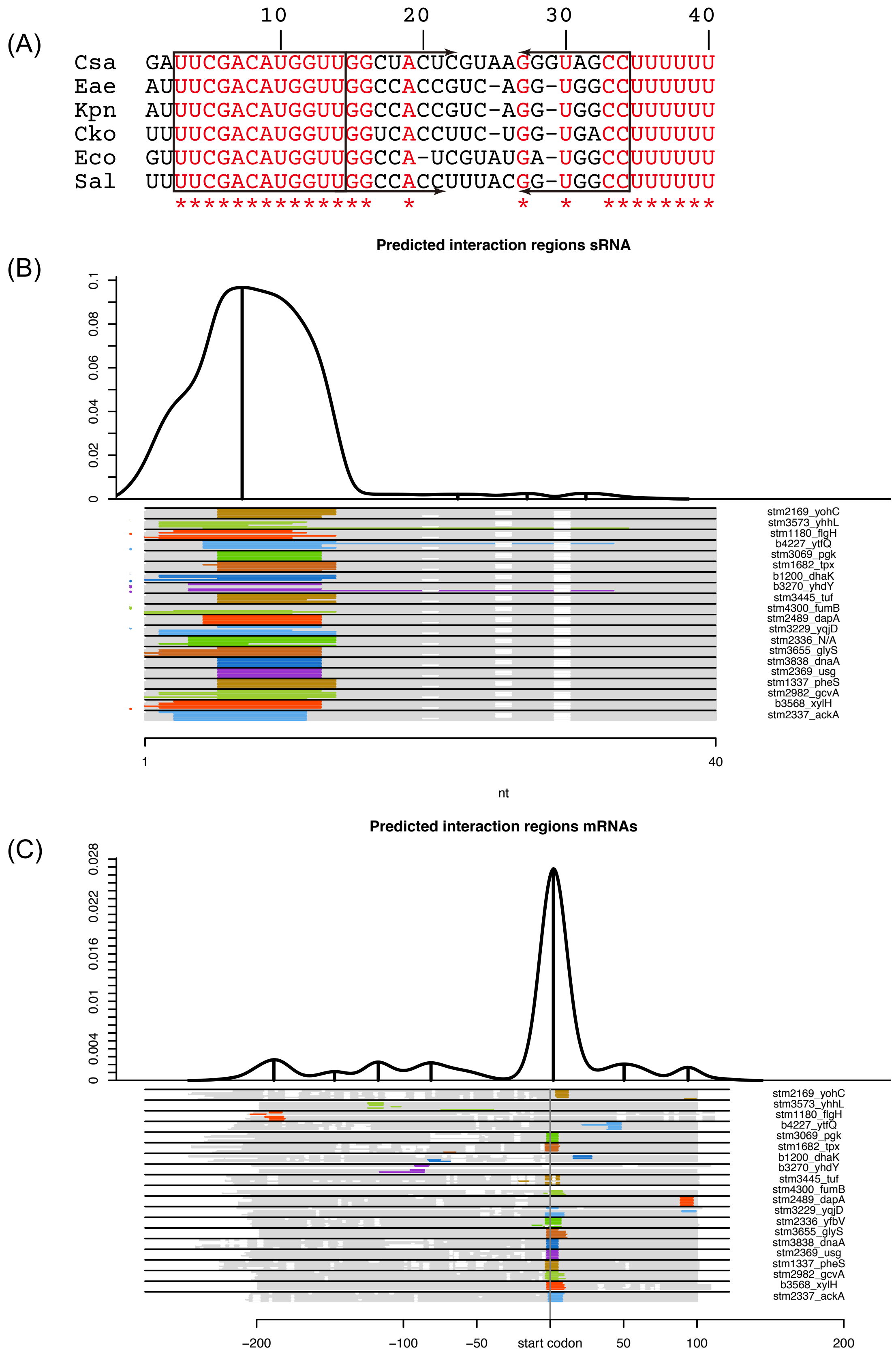

Fig. S2. Target prediction of SdhX from six Enterobacterial species by CopraRNA. (A) Alignment of the conserved 3' end region of SdhX. The stem-loop motif of Rho-independent terminator is indicated by inverted arrows. (B) Predicted interaction regions of SdhX. (C) Predicted interaction regions of candidate target mRNAs. Sal: Salmonella Typhimurium LT2 (NC_003197), Eco: E. coli MG1655 (NC_000913), Cko: Citrobacter koseri ATCC BAA-895 (NC_009792), Kpn: Klebsiella pneumoniae 342 (NC_011283), Eae: Enterobacter aerogenes KCTC 2190 (NC_015663), Csa: Cronobacter sakazakii ATCC BAA-894 (NC_009778). (D) Base pairing of putative target mRNAs with *Salmonella* SdhX2. Interactions of *Salmonella* SdhX2 with putative target mRNAsfrom RIL-seq data (Melamed et al., 2016).

Figure S2
Miyakoshi et al., 2018

(D)

ackA

```
             198        209
             |          |
5'-AUC...UACUU          UAAGU...UAG-3'
              CCAUGUCGAG
              |||||||||:
              GGUACAGCUU
3'-UUU...CGGUU          UU-5'
             |          |
             13         2
```

SdhX2

```
Energy                       -13.57 kcal/mol
Hybridization Energy         -15.5 kcal/mol
Unfolding Energy - mRNA      1.64163 kcal/mol
Unfolding Energy - sRNA      0.287387 kcal/mol

Position - mRNA              199 -- 208
Position - sRNA              3 -- 12
Position Seed - mRNA         202 -- 208
Position Seed - sRNA         3 -- 9
```

xylH (E. coli)

```
             197          210
             |            |
5'-GAA...CGAAC            AGCAA...UUG-3'
              AUCAUGUCGAAA
              |:||||||||||
              UGGUACAGCUUU
3'-UUU...CCGGU            G-5'
             |            |
             14           1
```

SdhX2

```
Energy                       -12.22 kcal/mol
Hybridization Energy         -15.7 kcal/mol
Unfolding Energy - mRNA      3.09399 kcal/mol
Unfolding Energy - sRNA      0.389078 kcal/mol

Position - mRNA              198 -- 209
Position - sRNA              2 -- 13
Position Seed - mRNA         203 -- 209
Position Seed - sRNA         2 -- 8
```

gcvA

```
             198     206
             |       |
5'-ACA...AGAGG       AAAAC...CGC-3'
              CCAUGUC
              |||||||
              GGUACAG
3'-UUU...CGGUU       CUUUU-5'
             |       |
             13      5
```

SdhX2

```
Energy                       -9.94 kcal/mol
Hybridization Energy         -11.7 kcal/mol
Unfolding Energy - mRNA      1.46776 kcal/mol
Unfolding Energy - sRNA      0.287386 kcal/mol

Position - mRNA              199 -- 205
Position - sRNA              6 -- 12
Position Seed - mRNA         199 -- 205
Position Seed - sRNA         6 -- 12
```

pheS

```
             196       206
             |         |
5'-CAC...AGGAA         ACAUC...GCA-3'
              AACCAUGUC
              |||||||||
              UUGGUACAG
3'-UUU...ACCGG         CUUUU-5'
             |         |
             15        5
```

SdhX2

```
Energy                       -11.45 kcal/mol
Hybridization Energy         -14 kcal/mol
Unfolding Energy - mRNA      2.26266 kcal/mol
Unfolding Energy - sRNA      0.287386 kcal/mol

Position - mRNA              197 -- 205
Position - sRNA              6 -- 14
Position Seed - mRNA         199 -- 205
Position Seed - sRNA         6 -- 12
```

usg

```
             197      206
             |        |
5'-AUU...AAAUC        UGAAG...AAA-3'
              ACCAUGUC
              ||||||||
              UGGUACAG
3'-UUU...CCGGU        CUUUU-5'
             |        |
             14       5
```

SdhX2

```
Energy                       -10.35 kcal/mol
Hybridization Energy         -12.4 kcal/mol
Unfolding Energy - mRNA      1.7644 kcal/mol
Unfolding Energy - sRNA      0.287386 kcal/mol

Position - mRNA              198 -- 205
Position - sRNA              6 -- 13
Position Seed - mRNA         199 -- 205
Position Seed - sRNA         6 -- 12
```

dnaA

```
             197      206
             |        |
5'-AUC...AGUCC        ACUUU...UGA-3'
              GCCGUGUC
              :||:||||
              UGGUACAG
3'-UUU...CCGGU        CUUUU-5'
             |        |
             14       5
```

SdhX2

```
Energy                       -10.92 kcal/mol
Hybridization Energy         -13.1 kcal/mol
Unfolding Energy - mRNA      1.89009 kcal/mol
Unfolding Energy - sRNA      0.287386 kcal/mol

Position - mRNA              198 -- 205
Position - sRNA              6 -- 13
Position Seed - mRNA         199 -- 205
Position Seed - sRNA         6 -- 12
```

glyS

```
             197            212
             |              |
5'-CGC...AGGCG        U     AACUU...CGG-3'
              GCCAUGUC GAGAA
              :||||||| ||:||
              UGGUACAG CUUUU
3'-UUU...CCGGU              -5'
             |              |
             14             1
```

SdhX2

```
Energy                       -11.92 kcal/mol
Hybridization Energy         -15.3 kcal/mol
Unfolding Energy - mRNA      3.0905 kcal/mol
Unfolding Energy - sRNA      0.287387 kcal/mol

Position - mRNA              198 -- 211
Position - sRNA              1 -- 13
Position Seed - mRNA         199 -- 205
Position Seed - sRNA         6 -- 12
```

yfbV

```
             196         208
             |           |
5'-UGC...AGGUA           CACCG...CCG-3'
              GGCCAUGUCGA
              ::|||||||||
              UUGGUACAGCU
3'-UUU...ACCGG           UUU-5'
             |           |
             15          3
```

SdhX2

```
Energy                       -12.64 kcal/mol
Hybridization Energy         -16.9 kcal/mol
Unfolding Energy - mRNA      3.97636 kcal/mol
Unfolding Energy - sRNA      0.287387 kcal/mol

Position - mRNA              197 -- 207
Position - sRNA              4 -- 14
Position Seed - mRNA         201 -- 207
Position Seed - sRNA         4 -- 10
```

yqjD

```
             196           210
             |             |
5'-CAG...GAGAA             GAUAA...AAG-3'
              AACUAUGUCGAAA
              |||:|||||||||
              UUGGUACAGCUUU
3'-UUU...ACCGG             U-5'
             |             |
             15            1
```

SdhX2

```
Energy                       -12.41 kcal/mol
Hybridization Energy         -16 kcal/mol
Unfolding Energy - mRNA      3.29862 kcal/mol
Unfolding Energy - sRNA      0.287387 kcal/mol

Position - mRNA              197 -- 209
Position - sRNA              2 -- 14
Position Seed - mRNA         203 -- 209
Position Seed - sRNA         2 -- 8
```

dapA

```
             288       298
             |         |
5'-GCC...UGAUU         CCA-3'
              AUCAUGUCG
              |:|||||||
              UGGUACAGC
3'-UUU...CCGGU         UUUU-5'
             |         |
             14        4
```

SdhX2

```
Energy                       -9.04 kcal/mol
Hybridization Energy         -11.4 kcal/mol
Unfolding Energy - mRNA      2.06902 kcal/mol
Unfolding Energy - sRNA      0.287386 kcal/mol

Position - mRNA              289 -- 297
Position - sRNA              5 -- 13
Position Seed - mRNA         291 -- 297
Position Seed - sRNA         5 -- 11
```

fumB

```
             199         211
             |           |
5'-GGC...AAGCA           AAGAG...AAU-3'
              CAUGUCGAAAA
              |||||||||||
              GUACAGCUUUU
3'-UUU...GGUUG           -5'
             |           |
             12          1
```

SdhX2

```
Energy                       -12.98 kcal/mol
Hybridization Energy         -15.4 kcal/mol
Unfolding Energy - mRNA      2.12788 kcal/mol
Unfolding Energy - sRNA      0.287387 kcal/mol

Position - mRNA              200 -- 210
Position - sRNA              1 -- 11
Position Seed - mRNA         204 -- 210
Position Seed - sRNA         1 -- 7
```

tufA

```
             196       206
             |         |
5'-AUG...AAUAU         UAAAG...CUA-3'
              AGCCGUGUC
              |:||:||||
              UUGGUACAG
3'-UUU...ACCGG         CUUUU-5'
             |         |
             15        5
```

SdhX2

```
Energy                       -9.12 kcal/mol
Hybridization Energy         -13 kcal/mol
Unfolding Energy - mRNA      3.59124 kcal/mol
Unfolding Energy - sRNA      0.287386 kcal/mol

Position - mRNA              197 -- 205
Position - sRNA              6 -- 14
Position Seed - mRNA         199 -- 205
Position Seed - sRNA         6 -- 12
```

Figure S3
Miyakoshi et al., 2018

ackA

```
            221        232
             |          |
5'-GCC...UACUU          UAAGU...AUC-3'
             CCAUGUCGAG
             |||||||||:
             GGUACAGCUU
3'-UUU...CGGUU          UGGCU...UUG-5'
             |          |
SdhX         86         75

Energy                       -10.21600 kcal/mol
Hybridization Energy         -15.3
Unfolding Energy - Target    1.72748
Unfolding Energy - Query     3.33787

Position - Target RNA        222 -- 231
Position - Query RNA         76 -- 85
Position Seed - Target RNA   225 -- 231
Position Seed - Query RNA    76 -- 82
```

fdoG

```
          113              128
           |                |
5'-ACC...GACGU   A          AAGGA...AAG-3'
              GAC AUGUCGAAAC
              :|| ||||||||||
              UUG UACAGCUUUG
3'-UUU...ACCGG   G          GCUGU...UUG-5'
           |                |
SdhX       88               73

Energy                       -7.04154 kcal/mol
Hybridization Energy         -16.3
Unfolding Energy - Target    4.24841
Unfolding Energy - Query     5.01005

Position - Target RNA        114 -- 127
Position - Query RNA         74 -- 87
Position Seed - Target RNA   121 -- 127
Position Seed - Query RNA    74 -- 80
```

katG

```
              3              18
              |              |
     5'-ACA                  ACAUU...CAA-3'
           CUGUAGAGGGGAGC
           ||||||||||:|||
           GACAUCUCCCUUCG
3'-UUU...GUAAG               CCGCU...UUG-5'
              |              |
SdhX          55             40

Energy                       -17.23490 kcal/mol
Hybridization Energy         -28.6
Unfolding Energy - Target    4.56325
Unfolding Energy - Query     6.80180

Position - Target RNA        4 -- 17
Position - Query RNA         41 -- 54
Position Seed - Target RNA   11 -- 17
Position Seed - Query RNA    41 -- 47
```

yehS

```
            115          127
             |            |
5'-CUU...GGGUA            ACCGC...UUU-3'
             AUGUCGAAGCC
             ||||||||:||
             UACAGCUUUGG
3'-UUU...GUUGG            CUGUC...UUG-5'
             |            |
SdhX         84           72

Energy                       -11.62110 kcal/mol
Hybridization Energy         -18.3
Unfolding Energy - Target    1.67041
Unfolding Energy - Query     5.00845

Position - Target RNA        116 -- 126
Position - Query RNA         73 -- 83
Position Seed - Target RNA   120 -- 126
Position Seed - Query RNA    73 -- 79
```

mnmA

```
           27                44
           |                 |
5'-GCC...GUGAU  A     U      CAAAA...UGG-3'
              CC AUGUC GAAACCG
              || ||||| |||||||
              GG UACAG CUUUGGC
3'-UUU...CGGUU               UGUCG...UUG-5'
           |                 |
SdhX       86                71

Energy                       -9.95006 kcal/mol
Hybridization Energy         -16.6
Unfolding Energy - Target    3.22778
Unfolding Energy - Query     3.46264

Position - Target RNA        28 -- 43
Position - Query RNA         72 -- 85
Position Seed - Target RNA   37 -- 43
Position Seed - Query RNA    72 -- 78
```

gdhA

```
              96                     118
              |                      |
5'-GCA...UCCUC         CAA    G      CCGAA...GAA-3'
              AACCAUGUC   AAGC CGAC
              |||||||||   ||:| ||||
              UUGGUACAG   UUUG GCUG
3'-UUU...ACCGG         C             UCGGG...UUG-5'
              |                      |
SdhX          88                     69

Energy                       -10.27590 kcal/mol
Hybridization Energy         -19.6
Unfolding Energy - Target    2.66239
Unfolding Energy - Query     6.66174

Position - Target RNA        97 -- 117
Position - Query RNA         70 -- 87
Position Seed - Target RNA   110 -- 117
Position Seed - Query RNA    70 -- 76
```

rcsF

```
             75      83
             |       |
5'-UUU...CUGUU       AAGCAG-3'
             CCAUGUU
             ||||||:
             GGUACAG
3'-UUU...CGGUU       CUUUG...UUG-5'
             |       |
SdhX         86      78

Energy                         -5.48976 kcal/mol
Hybridization Energy         -8.8
Unfolding Energy - Target    0.23222
Unfolding Energy - Query     3.07802

Position - Target RNA        76 -- 82
Position - Query RNA         79 -- 85
Position Seed - Target RNA   76 -- 82
Position Seed - Query RNA    79 -- 85
```

ftnA

```
           31              46
           |               |
5'-ACA...GCACU      C      AGAAA...GCC-3'
              AUCAUG UGAAACC
              |:|||| :||||||
              UGGUAC GCUUUGG
3'-UUU...CCGGU      A      CUGUC...UUG-5'
           |               |
SdhX       87              72

Energy                       -5.48387 kcal/mol
Hybridization Energy         -15.9
Unfolding Energy - Target    5.35052
Unfolding Energy - Query     5.06561

Position - Target RNA        32 -- 45
Position - Query RNA         73 -- 86
Position Seed - Target RNA   39 -- 45
Position Seed - Query RNA    73 -- 79
```

fdnG

```
             752      761
              |        |
5'-UCG...AGCAA         GGAUG...CAU-3'
              CGAAACCG
              ||||||||
              GCUUUGGC
3'-UUU...GUACA         UGUCG...UUG-5'
              |        |
SdhX          80       71

Energy                         -5.39535 kcal/mol
Hybridization Energy         -10.8
Unfolding Energy - Target    3.39938
Unfolding Energy - Query     2.04849

Position - Target RNA        753 -- 760
Position - Query RNA         72 -- 79
Position Seed - Target RNA   754 -- 760
Position Seed - Query RNA    72 -- 78
```

Fig. S3. Base pairing of putative target mRNAs with *E. coli* SdhX. Interactions of *E. coli* SdhX with putative target mRNAs from RIL-seq data (Melamed et al., 2016) were predicted by IntaRNA program (Mann et al., 2017).

Figure S4
Miyakoshi et al., 2018

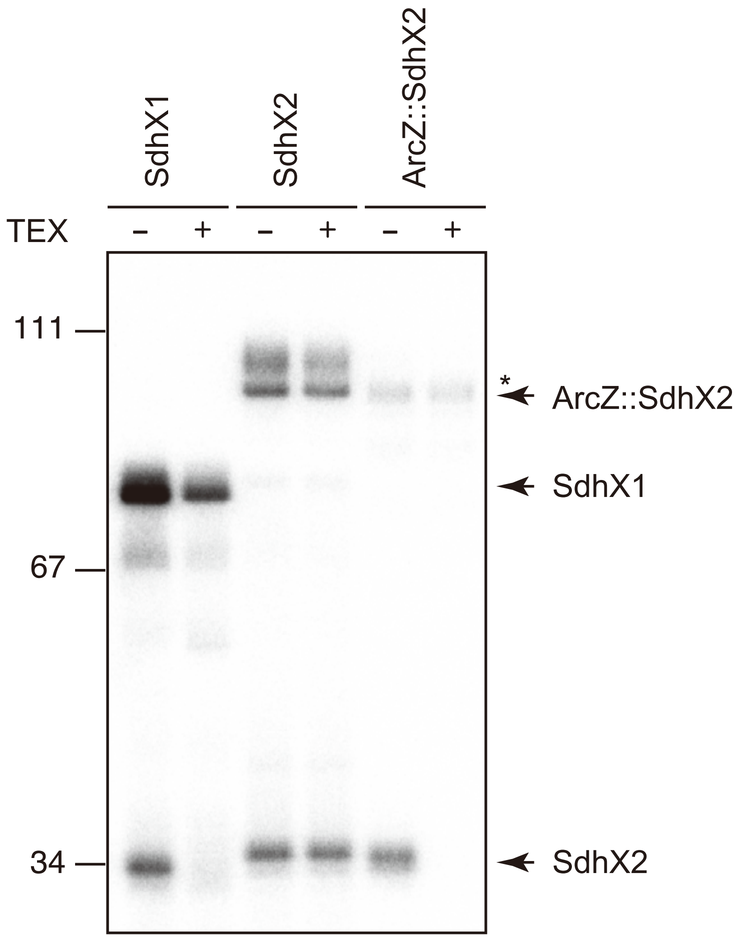

Fig. S4. 5’ -end status of artificially expressed SdhXD.

Total RNA was extracted from *Salmonella* Δ*sdhX* strains harboring expression plasmids of SdhX1 and SdhX2 (pLM1, pLM30, and pLM32, respectively), and treated with terminator 5’ -phosphate-dependent exonuclease (TEX; Epicenter). 5 μg of pretreated (-) and TEX-treated (+) total RNAs were analyzed by Northern blot. Asterisk indicates a putative read-through product, whose transcription terminates at downstream *rrnT1* in the vector. The size is estimated by pUC19 MspI dsDNA fragments.

Figure S5
Miyakoshi et al., 2018

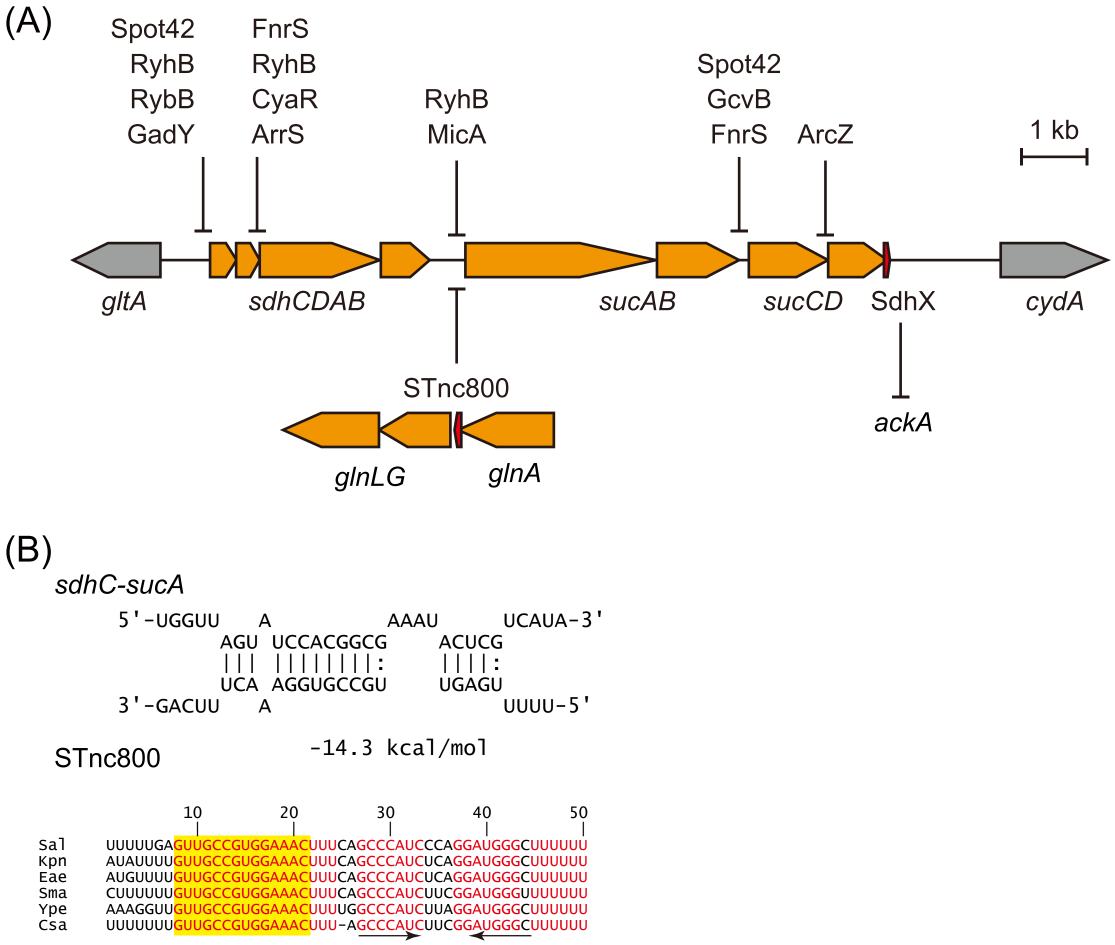

Fig. S5. Extended functions of metabolic mRNAs. (A) sdhCDAB-sucABCD operon mRNA as a hub for Hfq-dependent sRNAs. Mutiple sRNAs repress expression of TCA cycle enzymes while SdhX derived from 3' end of the operon mRNA represses multiple mRNAs. (B) Alignment of glnA 3' UTR and its predicted interaction with a target mRNA. Putative seed sequence is highlighted by yellow. The Rho-independent terminator is indicated by inverted arrows.

Figure S6
Miyakoshi et al., 2018

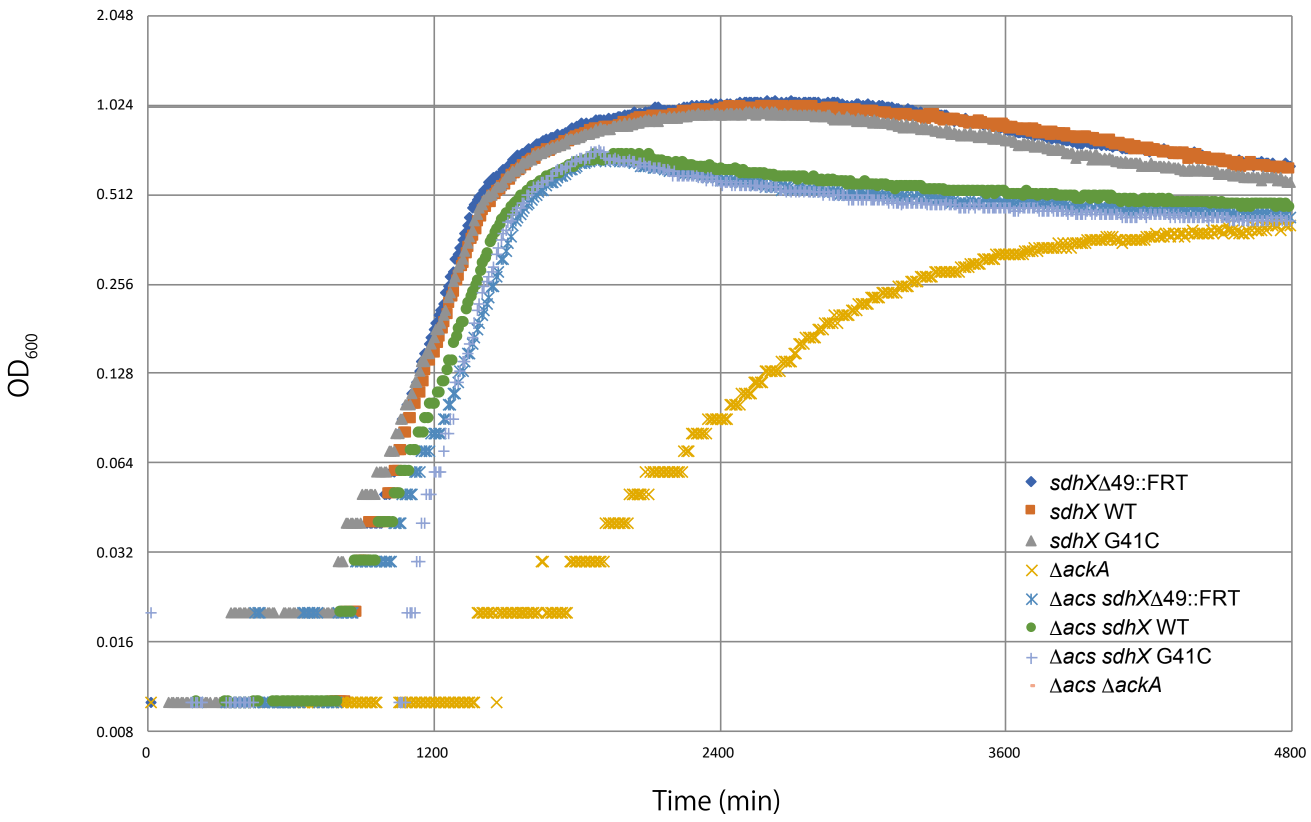

Fig. S6. Growth of *Salmonella* strains in MOPS medium supplemented with 40 mM acetate as the sole carbon source. Strains are listed in Supplemantary Table S1. Growth on acetate was significantly impaired by Δ*ackA* mutation and completely abolished by Δ*acs* Δ*ackA* double mutation.

Supplementary Table S1

Bacterial strains used in this study

Strain	Relevant markers/ genotype	Reference/ source
Salmonella enterica serovar Typhimurium		
JVS-1574	SL1344 Str^R *hisG rpsL xyl*	provided by D. Bumann
JVS-0673	Δ*rpoS*::Km	provided by K. Tedin
JVS-1227	Δ*arcA*::Km	Lab stock
JVS-1626	*crp773*::Tn*10* eutD::MudA	provided by M. Teplitski
JVS-6999	(*rluC*-*rne*)::Cm^R	(Figueroa-Bossi et al. 2009)
JVS-7000	(*rluC*-*rne*)::Cm^R *rne3071*	(Figueroa-Bossi et al. 2009)
JVS-10999	(*rluC*-*rne*)::Cm^R *rneR169K*	(Miyakoshi et al. 2015)
JVS-11058	Δ*sdhX*::Cm^R I-SceI	This study
JVS-11244	*sdhX*G41C	This study
JVS-11249	*ackA*-3xFLAG::Km^R	This study
JVS-11250	*sdhX*G41C *ackA*-3xFLAG::Km^R	This study
MMS-0001	*sdhX*Δ49::Km^R	This study
MMS-0003	*sdhX*Δ49::FRT	This study
MMS-0005	*sdhX*Δ49::FRT *ackA*-3xFLAG::Km^R	This study
MMS-0006	Δ*acs*::Cm *sdhX*Δ49::FRT	This study
MMS-0007	Δ*acs*::Cm	This study
MMS-0008	Δ*acs*::Cm *sdhX*G41C	This study
MMS-0009	Δ*ackA*::Km	This study
MMS-0010	Δ*ackA*::Km Δ*acs*::Cm	This study
MMS-0014	*sdhX*Δ49::FRT *ackA*-3xFLAG::FRT Δ*arcA*::Km	This study
MMS-0015	*ackA*-3xFLAG::FRT Δ*arcA*::Km	This study
Escherichia coli K-12		
BW25113	F^- λ^- *Δ(araD-B)567 Δ(rhaD-B)568 ΔlacZ4787(::rrnB-3) hsdR514 rph-1*	
MMS-0002	*sdhX*Δ75::Km^R	This study
MMS-0004	*sdhX*Δ75::FRT	This study

Supplementary Table S2

Plasmids used in this study

Name	Relevant fragment	Comment	Origin / marker	Reference
pZE12-luc	$P_{Llac\text{-}O}$-*luc*	Vector for sRNA overexpression, ColE1 origin	ColE1 / Ap^R	(Urban and Vogel 2007)
pJV300	$P_{Llac\text{-}O}$	Control plasmid, ColE1 origin	ColE1 / Ap^R	(Sittka et al. 2007)
pJU-19	$P_{Llac\text{-}O}$-*arcZ*	*Salmonella arcZ* expression plasmid, ColE1 origin	ColE1 / Ap^R	(Papenfort et al. 2009)
pLM1	$P_{Llac\text{-}O}$-*sdhX1*	*Salmonella sdhX1* was amplified with JVO-7189/JVO-5376, digested with XbaI, and cloned into pZE12-luc	ColE1 / Ap^R	This study
pLM29	$P_{Llac\text{-}O}$-*sdhX1* G41C	pLM1 derivative amplified with JVO-13326/JVO-13327	ColE1 / Ap^R	This study
pLM30	$P_{Llac\text{-}O}$-*sdhX2*	pLM1 derivative amplified with JVO-12421/JVO-13329	ColE1 / Ap^R	This study
pLM31	$P_{Llac\text{-}O}$-*sdhX2* G41C	pLM1 derivative amplified with JVO-13328/JVO-13329	ColE1 / Ap^R	This study
pLM32	$P_{Llac\text{-}O}$-*arcZ*::*sdhX2*	*Salmonella sdhX2* was amplified with JVO-12421/JVO-5376, digested with XbaI, and cloned into pJU-19 amplified by JVO-8976/JVO-8973	ColE1 / Ap^R	This study
pLM34	$P_{Llac\text{-}O}$-*sdhX1* CCC	pLM1 derivative amplified with JVO-13542/PLlacOC	ColE1 / Ap^R	This study
pLM35	$P_{Llac\text{-}O}$-*sdhX2* CCC	pLM1 derivative amplified with JVO-13562/JVO-8862	ColE1 / Ap^R	This study
pLM48	$P_{Llac\text{-}O}$-*sdhXeco*	*E. coli sdhX1* was amplified with JVO-7499/JVO-13379, digested with XbaI, and cloned into pZE12-luc	ColE1 / Ap^R	This study
pLM49	$P_{Llac\text{-}O}$-*sdhXeco* G67C	pLM48 derivative amplified with MMO-0147/MMO-0148	ColE1 / Ap^R	This study
pLM50	$P_{Llac\text{-}O}$-*sdhXeco* G62U	pLM48 derivative amplified with MMO-0167/MMO-0168	ColE1 / Ap^R	This study
pLM51	$P_{Llac\text{-}O}$-*sdhXeco* G62C	pLM48 derivative amplified with MMO-0190/MMO-0191	ColE1 / Ap^R	This study
pLM52	$P_{Llac\text{-}O}$-*sdhXeco* C34G	pLM48 derivative amplified with MMO-0232/MMO-0233	ColE1 / Ap^R	This study
pXG1	$P_{tet\text{-}O}$-*luc*RBS::*gfp*	Control vector containing GFP+	pSC101* / Cm^R	(Urban and Vogel 2007)

pXG1-sf	$P_{tet\text{-}O}$-*luc*RBS::*sfgfp*	Control vector containing superfolder GFP (sfGFP)	pSC101* / Cm^R	This study
pXG10-sf	$P_{tet\text{-}O}$-*sfgfp*	Vector containing sfGFP for standard translational fusion	pSC101* / Cm^R	(Corcoran et al. 2012)
pXG30-sf	$P_{tet\text{-}O}$-*FLAG::lacZ'-sfgfp*	Vector containing sfGFP for intraoperonic translational fusion	pSC101* / Cm^R	(Corcoran et al. 2012)
pXG10-ackAsal	$P_{tet\text{-}O}$-*ackA::sfgfp*	*Salmonella ackA* 12aa was amplified with JVO-13376/JVO-13377, digested with NsiI/NheI, and cloned into pXG10-sf	pSC101* / Cm^R	This study
pXG10-ackAeco	$P_{tet\text{-}O}$-*ackA::sfgfp*	*E. coli ackA* 12aa was amplified with MMO-0081/JVO-13377, digested with NsiI/NheI, and cloned into pXG10-sf	pSC101* / Cm^R	This study
pXG30-fumBsal	$P_{tet\text{-}O}$- *FLAG::lacZ'::dcuB-fumB::sfgfp*	*Salmonella dcuB* 226aa-*fumB* 20aa was amplified with MMO-0193/JVO-12156, digested with NsiI/NheI, and cloned into pXG30-sf	pSC101* / Cm^R	This study
pXG30-fumBeco	$P_{tet\text{-}O}$- *FLAG::lacZ'::dcuB-fumB::sfgfp*	*E. coli dcuB* 226aa-*fumB* 20aa was amplified with MMO-0193/MMO-0056, digested with NsiI/NheI, and cloned into pXG30-sf	pSC101* / Cm^R	This study
pXG30-xylGHeco	$P_{tet\text{-}O}$- *FLAG::lacZ'::xylG-xylH::sfgfp*	*E. coli xylG* 80aa-*xylH* 20aa was amplified with MMO-0194/0195, digested with NsiI/NheI, and cloned into pXG30-sf	pSC101* / Cm^R	This study
pXG10-fdoGeco	$P_{tet\text{-}O}$-*fdoG::sfgfp*	*E. coli fdoG* 10aa was amplified with MMO-0126/MMO-0127, digested with NsiI/NheI, and cloned into pXG10-sf	pSC101* / Cm^R	This study
pXG10-fdoGsal	$P_{tet\text{-}O}$-*fdoG::sfgfp*	*Salmonella fdoG* 10aa was amplified with MMO-0145/MMO-0127, digested with NsiI/NheI, and cloned into pXG10-sf	pSC101* / Cm^R	This study
pXG10-gdhAeco	$P_{tet\text{-}O}$-*gdhA::sfgfp*	*E. coli gdhA* 40aa was amplified with MMO-0199/MMO-0210, digested with NsiI/NheI, and cloned into pXG10-sf	pSC101* / Cm^R	This study
pXG10-katGeco	$P_{tet\text{-}O}$-*katG::sfgfp*	*E. coli katG* 40aa was amplified with MMO-0211/ MMO-0212, digested with NsiI/NheI, and cloned into pXG10-sf	pSC101* / Cm^R	This study
pXG10-yehSeco	$P_{tet\text{-}O}$-*yehS::sfgfp*	*E. coli yehS* 50aa was amplified with MMO-0215/ MMO-0216, digested with NsiI/NheI, and cloned into pXG10-sf	pSC101* / Cm^R	This study
pXG10-rcsFeco	$P_{tet\text{-}O}$-*rcsF::sfgfp*	*E. coli rcsF* 24aa was amplified with MMO-0217/ MMO-0218, digested with NsiI/NheI, and cloned into pXG10-sf	pSC101* / Cm^R	This study
pXG10-mnmAeco	$P_{tet\text{-}O}$-*mnmA::sfgfp*	*E. coli mnmA* 30aa was amplified with MMO-0230/ MMO-0231, digested with NsiI/NheI, and cloned into pXG10-sf	pSC101* / Cm^R	This study

pXG10-dnaAsal	$P_{tet\text{-}O}$-*dnaA::sfgfp*	*Salmonella dnaA* 20aa was amplified with MMO-0240/ MMO-0241, digested with NsiI/NheI, and cloned into pXG10-sf	pSC101* / Cm^R	This study
pXG10-yfbVsal	$P_{tet\text{-}O}$-*yfbV::sfgfp*	*Salmonella yfbV* 10aa was amplified with MMO-0242/ MMO-0243, digested with NsiI/NheI, and cloned into pXG10-sf	pSC101* / Cm^R	This study
pXG10-dapAsal	$P_{tet\text{-}O}$-*dapA::sfgfp*	*Salmonella dapA* 40aa was amplified with MMO-0244/ MMO-0245, digested with NsiI/NheI, and cloned into pXG10-sf	pSC101* / Cm^R	This study
pXG10-tufAsal	$P_{tet\text{-}O}$-*tufA::sfgfp*	*Salmonella tufA* 40aa was amplified with MMO-0246/ MMO-0247, digested with NsiI/NheI, and cloned into pXG10-sf	pSC101* / Cm^R	This study
pXG30-glyQSsal	$P_{tet\text{-}O}$- *FLAG::lacZ'::glyQ-glyS::sfgfp*	*Salmonella glyQ* 10aa-glyS 20aa was amplified with MMO-0248/ MMO-0249, digested with NsiI/NheI, and cloned into pXG30-sf	pSC101* / Cm^R	This study
pXG10-katGsal	$P_{tet\text{-}O}$-*katG::sfgfp*	*Salmonella katG* 40aa was amplified with MMO-0266/ MMO-0267, digested with NsiI/NheI, and cloned into pXG10-sf	pSC101* / Cm^R	This study
pXG30-yqjCDsal	$P_{tet\text{-}O}$- *FLAG::lacZ'::yqjC-yqjD::sfgfp*	*Salmonella yqjC* 40aa-yqjD 20aa was amplified with MMO-0279/ MMO-0280, digested with NsiI/NheI, and cloned into pXG30-sf	pSC101* / Cm^R	This study
pXG10-yfbVeco	$P_{tet\text{-}O}$-*yfbV::sfgfp*	*Salmonella yfbV* 10aa was amplified with MMO-0253/ MMO-0254, digested with NsiI/NheI, and cloned into pXG10-sf	pSC101* / Cm^R	This study
pXG10-ackAsal C5G	$P_{tet\text{-}O}$-*$ackA_{C5G}$::sfgfp*	pXG10-ackAsal derivative amplified by MMO-0203/MMO-0204	pSC101* / Cm^R	This study
pXG30-fumBsal C5G	$P_{tet\text{-}O}$- *FLAG::lacZ'::dcuB-fumB::sfgfp*	pXG30-fumBsal derivative amplified by MMO-0257/MMO-0258	pSC101* / Cm^R	This study
pXG30-fumBsal AC-1U	$P_{tet\text{-}O}$- *FLAG::lacZ'::dcuB-fumB::sfgfp*	pXG30-fumBsal derivative amplified by MMO-0156/ MMO-0157	pSC101* / Cm^R	This study
pXG30-fumBsal G6A	$P_{tet\text{-}O}$- *FLAG::lacZ'::dcuB-fumB::sfgfp*	pXG30-fumBsal derivative amplified by MMO-0158/ MMO-0159	pSC101* / Cm^R	This study
pXG30-fumBsal AC-1U/G6A	$P_{tet\text{-}O}$- *FLAG::lacZ'::dcuB-fumB::sfgfp*	pXG30-fumBsal derivative amplified by MMO-0158/ MMO-0160	pSC101* / Cm^R	This study
pXG30-fumBeco U-1AC	$P_{tet\text{-}O}$- *FLAG::lacZ'::dcuB-fumB::sfgfp*	pXG30-fumBeco derivative amplified by MMO-0058/MMO-0059	pSC101* / Cm^R	This study

pXG30-fumBeco A6G	$P_{tet\text{-}O}$- *FLAG::lacZ'::dcuB-fumB::sfgfp*	pXG30-fumBeco derivative amplified by MMO-0062/MMO-0063	pSC101* / Cm^R	This study
pXG30-fumBeco U-1AC/A6G	$P_{tet\text{-}O}$- *FLAG::lacZ'::dcuB-fumB::sfgfp*	pXG30-fumBeco derivative amplified by MMO-0062/MMO-0112	pSC101* / Cm^R	This study
pXG10-yfbVsal C5G	$P_{tet\text{-}O}$-*yfbV::sfgfp*	pXG10-yfbVsal derivative amplified by MMO-0255/MMO-0256	pSC101* / Cm^R	This study
pXG10-yfbVsal C-2G	$P_{tet\text{-}O}$-*yfbV::sfgfp*	pXG10-yfbVsal derivative amplified by MMO-0274/MMO-0275	pSC101* / Cm^R	This study
pXG10-yfbVsal G6A	$P_{tet\text{-}O}$-*yfbV::sfgfp*	pXG10-yfbVsal derivative amplified by MMO-0276/MMO-0277	pSC101* / Cm^R	This study
pXG10-yfbVsal C-2G/G6A	$P_{tet\text{-}O}$-*yfbV::sfgfp*	pXG10-yfbVsal derivative amplified by MMO-0276/MMO-0278	pSC101* / Cm^R	This study
pXG10-yfbVeco G-2C	$P_{tet\text{-}O}$-*yfbV::sfgfp*	pXG10-yfbVeco derivative amplified by MMO-0269/MMO-0270	pSC101* / Cm^R	This study
pXG10-yfbVeco A6G	$P_{tet\text{-}O}$-*yfbV::sfgfp*	pXG10-yfbVeco derivative amplified by MMO-0271/MMO-0272	pSC101* / Cm^R	This study
pXG10-yfbVeco G-2C/A6G	$P_{tet\text{-}O}$-*yfbV::sfgfp*	pXG10-yfbVeco derivative amplified by MMO-0271/MMO-0273	pSC101* / Cm^R	This study
pXG10-fdoGeco C-13G	$P_{tet\text{-}O}$-*fdoG::sfgfp*	pXG10-fdoGeco derivative MMO-0188/MMO-0189	pSC101* / Cm^R	This study
pXG10-katGeco G-12C	$P_{tet\text{-}O}$-*katG::sfgfp*	pXG10-katGeco derivative MMO-0234/MMO-0235	pSC101* / Cm^R	This study
pKD4		template of Km^R cassette	oriRγ / Ap^R	(Datsenko and Wanner 2000)
pKD46		temperature-sensitive lambda Red expression plasmid	oriR101 / Ap^R	(Datsenko and Wanner 2000)
pCP20		temperature-sensitive FLP expression plasmid	oriR101 / Ap^R	(Datsenko and Wanner 2000)
pWRG99		temperature-sensitive lambda Red and I-SceI expression plasmid	oriR101 / Ap^R	(Blank et al. 2011)
pWRG100		template of Cm^R I-SceI cassette	oriRγ / Ap^R	(Blank et al. 2011)

Supplementary Table S3

DNA oligonucleotides used in this study

Name	Sequence (5' to 3' direction)	Used for
JVO-0322	CTACGGCGTTTCACTTCTGAGTTC	Northern blot probe
JVO-5376	GTTTTTTCTAGAGCCACAAACAATAAAGC	sdhXsal cloning
JVO-7187	GGCCGATATCGGCGAAGCGCTGAAAGCAATTATAAAATAAGTGTAGGCTGG AGCTGCTTC	sdhXsal deletion
JVO-7189	P~ATTATAAAATAAAACTCGCTGTTCC	sdhXsal cloning
JVO-7499	CGTGAAAACCGTTCGCAGC	sdhXsal/eco cloning
JVO-8973	CAGTATTCGCGCACCCCG	arcZ chimera cloning
JVO-8976	P~ATCGTGGCTGCGCCGT	arcZ chimera cloning
JVO-12156	GTTTTTGCTAGCCTCTGTTTTGTCCTCTCC	fumBsal cloning
JVO-12240	AAACAATAAAGCCATAAAAAAGGCCACCGTAAAGGTGGCCCTAGACTATAT TACCCTGTT	sdhXsal deletion
JVO-12421	TTTCGACATGGTTGGC	sdhXsal cloning
JVO-13308	AGAACTGGTTATCGCGCAAGACGCGAGCCGTCTGACTGCCGACTACAAAGA CCATGACGG	ackAsal 3xFLAG tagging
JVO-13309	GGACGGGTTACAAAACAGCACCGCCAGCGAGGCTGGCGGTCCATATGAATA TCCTCCTTAG	ackAsal 3xFLAG tagging
JVO-13226	CGTTTTCCACATGGTTGGCCACCT	sdhXsal mutation
JVO-13227	ACCATGTGGAAAACGGACATTTATCTGTTC	sdhXsal mutation
JVO-13228	TTTCCACATGGTTGGCCAC	sdhXsal mutation
JVO-13229	CGTGCTCAGTATCTTGTTATCCG	sdhXsal mutation
JVO-13376	GTTTTTATGCATGCCTGTCAAAGGCCGG	ackAsal cloning
JVO-13377	GTTTTTGCTAGCACCGCAGTTCAGAACCAGTAC	ackAsal/eco cloning
JVO-13379	GTTTTTTCTAGAAAATCATCGCGATAAGC	sdhXeco cloning
JVO-13380	GGCGGATATCGGTGAAGCACTGAAAACTGTTCTGAAATAAGTGTAGGCTGG AGCTGCTTC	sdhXeco deletion
JVO-13542	AACCATGTCGAGGGCGGACATTTATCTGTTCCC	arcZ chimera cloning
JVO-13562	CCCTCGACATGGTTGGCCAC	arcZ chimera cloning
JVO-13619	GGATTCGAACCTCCGACCCACTGG	Northern blot probe
MMO-0056	GTTTTTGCTAGCTTCGGTATTGTCTTTCC	fumBeco cloning
MMO-0058	GAAGCACATGTCAAACAAACCCTTTAT	fumBeco mutation
MMO-0059	GACATGTGCTTCCAGCCTGTAACT	fumBeco mutation
MMO-0062	ATGTCGAACAAACCCTTTATCTACCA	fumBeco mutation
MMO-0063	TTGTTCGACATAGCTTCCAGCCT	fumBeco mutation
MMO-0079	AGAACTGGTTATCGCGCAAGACGCGAGCCGCCTGACTGCCGACTACAAAGA CCATGACGG	ackAeco 3xFLAG tagging
MMO-0080	TGGCGGGTTACAAAACAGCACCGCCAGCTGAGCTGGCGGTCCATATGAATA TCCTCCTTAG	ackAeco 3xFLAG tagging
MMO-0081	GTTTTTATGCATGCCTGAAGGCCTAAGTAGTAC	ackAeco cloning

MMO-0112	TTGTTCGACATGTGCTTCCAGC	fumBeco mutation
MMO-0126	ACTGAGCACATGCATACCCTTCCTGGATGGAG	fdoGeco cloning
MMO-0127	TTCTCCTTTGCTAGCCTTAAAGAACTGCCTTCTGCTG	fdoGsal/eco cloning
MMO-0145	ACTGAGCACATGCATACTCCTCCCGGATGGAG	fdoGsal cloning
MMO-0147	GGTTTCCACATGGTTGGCCATCG	sdhXeco mutation
MMO-0148	CCATGTGGAAACCGACAGCCCTTC	sdhXeco mutation
MMO-0156	GAAGCTATGTCGAAAAAAGAGTTTATCT	fumBsal mutation
MMO-0157	GACATAGCTTCCAGCCTAATAGTAAG	fumBsal mutation
MMO-0158	ATGTCAAAAAAAGAGTTTATCTATCAGGCG	fumBsal mutation
MMO-0159	TTTTTTGACATGTGCTTCCAGCC	fumBsal mutation
MMO-0160	TTTTTTGACATAGCTTCCAGCCTAATAG	fumBsal mutation
MMO-0167	CTGTCGTTTTCGACATGGTTGGCCATC	sdhXeco mutation
MMO-0168	TCGAAAACGACAGCCCTTCGCCAT	sdhXeco mutation
MMO-0183	TCATCGCGATAAGCACAAAAAAGGCCATCATACGATGGCCGGTCCATATGAATATCCTCCTTAG	sdhXeco deletion
MMO-0186	TCGAAAAAAGGAGCAATCCATGCAG	fdoGeco mutation
MMO-0187	CTCCTTTTTTCGACATTGTCACGTC	fdoGeco mutation
MMO-0188	TCGAAAGAAGGAGCAATCCATGCAG	fdoGeco mutation
MMO-0189	CTCCTTCTTTCGACATTGTCACGTC	fdoGeco mutation
MMO-0190	CTGTCGCTTTCGACATGGTTGGCCATC	sdhXeco mutation
MMO-0191	TCGAAAGCGACAGCCCTTCGCCAT	sdhXeco mutation
MMO-0199	GTTTTATGCATGCAAAAGCACATGACATA	gdhAeco cloning
MMO-0203	CCATGTGGAGTAAGTTAGTACTGGTTCTGAAC	ackAsal mutation
MMO-0204	TTACTCCACATGGAAGTACCTATTTATGATAC	ackAsal mutation
MMO-0210	GTTTTTGCTAGCTTCAAGAAAAGGCCAGAG	gdhAeco cloning
MMO-0211	GTTTTATGCATACACTGTAGAGGGGAGCAC	katGeco cloning
MMO-0212	GTTTTTGCTAGCTTGATTTGGCCACCAGT	katGeco cloning
MMO-0215	GTTTTTATGCATCTTTTTGCCTCATCAACA	yehSeco cloning
MMO-0216	GTTTTTGCTAGCCTGAAAACCCTCTTCGTC	yehSeco cloning
MMO-0217	GTTTTATGCATTTTGATCATACATTGAGGA	rcsFeco cloning
MMO-0218	GTTTTTGCTAGCGACAGGGGATCTGCTTAA	rcsFeco cloning
MMO-0230	GTTTTTATGCATGCCTTGAAGTTCAATGTC	mnmAeco cloning
MMO-0231	GTTTTTGCTAGCATATCCCTGTTGTTGCA	mnmAeco cloning
MMO-0232	CGCTTCGCTCTACAGGAATGGCGAAGGG	sdhXeco mutation
MMO-0233	GTAGAGCGAAGCGGCGAGGGCTATT	sdhXeco mutation
MMO-0234	GTAGAGCGGAGCACATTGATGAGCA	katGeco mutation
MMO-0235	TGCTCCGCTCTACAGTGTATGCATGTGCTC	katGeco mutation

MMO-0240	GTTTTTATGCATTTTCCTGGAAAGTCCTGT	dnaAsal cloning
MMO-0241	GTTTTTGCTAGCTGTGGCTGGTAACTCATC	dnaAsal cloning
MMO-0242	GTTTATGCATAATATTGACGACTCAATGAT	yfbVsal cloning
MMO-0243	GTTTTTGCTAGCATTTACGGAGCGATTATC	yfbVsal cloning
MMO-0244	GTTTTTATGCATACTTGTTTGCACAGAGGA	dapAsal cloning
MMO-0245	GTTTTTGCTAGCAACAATCGCCGAGGTAC	dapAsal cloning
MMO-0246	GTTTATGCATGTCTATCGCCTTTAAAGAAG	tufAsal cloning
MMO-0247	GTTTTGCTAGCGTAGGTTTTAGCCAGTACG	tufAsal cloning
MMO-0248	GTTTTTATGCATGGCTTCCCGATGTGCAAC	glyQSsal cloning
MMO-0249	GTTTTTGCTAGCGAGGCCGGCGTTATCCAG	glyQSsal cloning
MMO-0253	GTTTTTATGCATAATTTGACGACTCAATG	yfbVsal cloning
MMO-0254	GTTTTGCTAGCATTAACAGAACGATTATCC	yfbVsal cloning
MMO-0255	CCATGTGGACACCGGATAATCGCTC	yfbVsal mutation
MMO-0256	GGTGTCCACATGGCCTACCTCAACTTC	yfbVsal mutation
MMO-0257	CATGTGGAAAAAAGAGTTTATCTATCAGGC	fumBsal mutation
MMO-0258	TTTTCCACATGTGCTTCCAGCCTA	fumBsal mutation
MMO-0261	TCATCGCGATAAGCACAAAAAAGGCCATCATACGATGGCCCTAGACTATAT TACCCTGTT	sdhXeco deletion
MMO-0266	GTTTTATGCATACACTTTAAAAGGGAGCTGAGATATG	katGsal cloning
MMO-0267	GTTTTTGCTAGCCTGGTTCGGCCACCAGT	katGsal cloning
MMO-0269	GGTGAACCATGTCAACGCCGGATA	yfbVeco mutation
MMO-0270	GACATGGTTCACCTCAACTTCACATATAAA	yfbVeco mutation
MMO-0271	CATGTCGACGCCGGATAATCGTTC	yfbVeco mutation
MMO-0272	CGGCGTCGACATGCTTCACCTCAACTTC	yfbVeco mutation
MMO-0273	CGGCGTCGACATGGTTCACCTCAACTTC	yfbVeco mutation
MMO-0274	GGTAGGGCATGTCGACACCGGATA	yfbVsal mutation
MMO-0275	GACATGCCCTACCTCAACTTCACATATAAA	yfbVsal mutation
MMO-0276	CATGTCAACACCGGATAATCGCTC	yfbVsal mutation
MMO-0277	CGGTGTTGACATGGCCTACCTCAAC	yfbVsal mutation
MMO-0278	CGGTGTTGACATGCCCTACCTCAAC	yfbVsal mutation
MMO-0279	GTTTTTATGCATCGCCAACGTGATTTAGC	yqjCDsal cloning
MMO-0280	GTTTTTGCTAGCCGTATCGGTCAGGGATTT	yqjCDsal cloning
MMO-0294	ATATCTGTAATAAGAAATAGCCCTCG	sdhXeco cloning
MMO-0315	GTAAAGGTGGCCAACCATGT	Northern blot probe
MMO-0317	CCGTAAAGGTGGCCAACCATGTCG	Northern blot probe

"p~" denotes a 5' monophosphate modification.

Reference:

Blank K, Hensel M, Gerlach RG. 2011. Rapid and highly efficient method for scarless mutagenesis within the Salmonella enterica chromosome. *PLoS One* **6**: e15763.

Corcoran CP, Podkaminski D, Papenfort K, Urban JH, Hinton JC, Vogel J. 2012. Superfolder GFP reporters validate diverse new mRNA targets of the classic porin regulator, MicF RNA. *Mol Microbiol* **84**: 428-445.

Datsenko KA, Wanner BL. 2000. One-step inactivation of chromosomal genes in Escherichia coli K-12 using PCR products. *Proc Natl Acad Sci U S A* **97**: 6640-6645.

Figueroa-Bossi N, Valentini M, Malleret L, Fiorini F, Bossi L. 2009. Caught at its own game: regulatory small RNA inactivated by an inducible transcript mimicking its target. *Genes Dev* **23**: 2004-2015.

Melamed, S., Peer, A., Faigenbaum-Romm, R., Gatt, Y.E., Reiss, N., Bar, A., Altuvia, Y., Argaman, L. and Margalit, H. (2016) Global Mapping of Small RNA-Target Interactions in Bacteria. *Mol Cell*, **63**, 884-897.

Miyakoshi M, Chao Y, Vogel J. 2015. Cross talk between ABC transporter mRNAs via a target mRNA-derived sponge of the GcvB small RNA. *EMBO J* **34**: 1478-1492.

Papenfort K, Said N, Welsink T, Lucchini S, Hinton JC, Vogel J. 2009. Specific and pleiotropic patterns of mRNA regulation by ArcZ, a conserved, Hfq-dependent small RNA. *Mol Microbiol* **74**: 139-158.

Sittka A, Pfeiffer V, Tedin K, Vogel J. 2007. The RNA chaperone Hfq is essential for the virulence of Salmonella typhimurium. *Mol Microbiol* **63**: 193-217.

Urban JH, Vogel J. 2007. Translational control and target recognition by Escherichia coli small RNAs in vivo. *Nucleic Acids Res* **35**: 1018-1037.
